# Geological Changes of the Americas and their Influence on the Diversification of the Neotropical Kissing Bugs (Hemiptera: Reduviidae: Triatominae)

**DOI:** 10.1371/journal.pntd.0004527

**Published:** 2016-04-08

**Authors:** Silvia A. Justi, Cleber Galvão, Carlos G. Schrago

**Affiliations:** 1 Departamento de Genética, Universidade Federal do Rio de Janeiro, Brazil; 2 Laboratório Nacional e Internacional de Referência em Taxonomia de Triatomíneos, Instituto Oswaldo Cruz, FIOCRUZ, Rio de Janeiro, Brazil; Temple University, UNITED STATES

## Abstract

**Background:**

The family Reduviidae (Hemiptera: Heteroptera), or assassin bugs, is among the most diverse families of the true bugs, with more than 6,000 species. The subfamily Triatominae (kissing bugs) is noteworthy not simply because it is the only subfamily of the Reduviidae whose members feed on vertebrate blood but particularly because all 147 known members of the subfamily are potential Chagas disease vectors. Due to the epidemiological relevance of these species and the lack of an efficient treatment and vaccine for Chagas disease, it is more common to find evolutionary studies focusing on the most relevant vectors than it is to find studies aiming to understand the evolution of the group as a whole. We present the first comprehensive phylogenetic study aiming to understand the events that led to the diversification of the Triatominae.

**Methodology/Principal Findings:**

We gathered the most diverse samples of Reduviidae and Triatominae (a total of 229 Reduviidae samples, including 70 Triatominae species) and reconstructed a robust dated phylogeny with several fossil (Reduviidae and Triatominae) calibrations. Based on this information, the possible role of geological events in several of the major cladogenetic events within Triatominae was tested for the first time. We were able to not only correlate the geological changes in the Neotropics with Triatominae evolution but also add to an old discussion: Triatominae monophyly *vs*. paraphyly.

**Conclusions/Significance:**

We found that most of the diversification events observed within the Rhodniini and Triatomini tribes are closely linked to the climatic and geological changes caused by the Andean uplift in South America and that variations in sea levels in North America also played a role in the diversification of the species of *Triatoma* in that region.

## Author Summary

We present the first comprehensive phylogenetic study aiming to understand the geological events that led to Triatominae diversification. We gathered the most diverse sampling of Reduviidae and Triatominae to construct a robust and accurate phylogeny and to be able to use more than the two Triatominae fossils as calibration points for our time estimates. The possible role of geological events in several of the major cladogenetic events within Triatominae is tested for the first time in the present study. We were able not only to correlate the geological changes in the Neotropics with Triatominae evolution but also to add to an old discussion: Triatominae monophyly vs. paraphyly.

## Introduction

The family Reduviidae (Hemiptera: Heteroptera), or assassin bugs, is among the most diverse of the true bugs (Heteroptera) [1]. A great variety of predatory behaviour can be observed within this group of over 6,000 species [1]. The subfamily Triatominae (kissing bugs) is noteworthy not simply as the only subfamily of the Reduviidae whose species feed on vertebrate blood [2] but especially as the vector of Chagas disease. All 147 known members of the Triatominae are believed to be potential Chagas disease vectors [3]. This illness is caused by contact between the faeces of vectors infected with the protozoan *Trypanosoma cruzi* and the blood and mucosa of vertebrates [4] and is a major public health and economic problem in Latin America [5].

The Triatominae are divided into five tribes: Alberproseniini, Bolboderini, Cavernicolini, Rhodniini and Triatomini [6], which are distributed from the southern USA to Patagonia, with a few species of Triatomini known from India and Australia [3]. The first three tribes comprise only 15 out of the 147 known species. Rhodniini and Triatomini are the most diverse and epidemiologically relevant tribes and are, therefore, the best studied.

Due to the epidemiological relevance of the Triatominae and the lack of an efficient treatment and vaccine for Chagas disease, it is more common to find evolutionary studies focusing on the most relevant vectors rather than studies aiming to understand the evolution of the group as a whole [7]. Even the studies that included the greatest diversity of species in the subfamily [8–11] did not seek to understand the events behind the diversification, only the relationships among the species.

The most diverse genera within the Rhodniini (*Rhodnius*) and Triatomini (*Triatoma*) have classically been divided into subgroups, primarily based on morphology and geographical distribution [6], although the paraphyly of *Triatoma* has long been known [10]. Only the Rhodniini tribe has been the focus of area biogeography studies within Triatominae [5,12], although neither of these studies formally tested the influence of the hypothesised geological events on the lineages studied or even presented a dated phylogeny for these lineages.

To date, the number of molecular dating studies involving Triatominae species is extremely restricted [2,13,14], but the times estimated for the origin of the subfamily differ by approximately 70 Myr. Although no previous studies have aimed to unravel the connection between the geological changes in the Americas during the late Palaeogene and Neogene Periods and Triatominae diversification, a close association between monophyletic groups within Triatomini and biogeographically defined areas has already been observed [11].

This paper presents the first comprehensive phylogenetic study aiming to understand the events that led to Triatominae diversification. We gathered the most diverse sampling of Reduviidae [2] and Triatominae [11] to construct a robust and accurate phylogeny [15–19] and to be able to use more than the two Triatominae fossils [20,21] as calibration points for our time estimates. The possible role of geological events in several of the major cladogenetic events within Triatominae is tested for the first time in the present study. We were able not only to correlate the geological changes in the Neotropics with Triatominae evolution but also to add to an old discussion: Triatominae monophyly *vs*. paraphyly.

## Methods

### Taxon Sampling

To increase accuracy [15–19] and to be able to use more fossil calibrations, we included all Reduviidae and the outgroups used by Hwang & Weirauch [2] representative of 18 Reduviidae subfamilies out of the 25 recognised for Reduviidae, totalling 159 species. To investigate divergence times in Triatominae lineages, we used 11 out of the 22 described Rhodniini species, representative of both genera (*Rhodnius* and *Psammolestes*) assigned to this tribe and of the three *Rhodnius* species groups. Representing the Triatomini tribe, 57 species were included, representing four of the accepted genera and all the *Triatoma* complexes, as reviewed by Schofield & Galvão [6]. Additionally, *Cavernicola pilosa*, representative of the rare Cavernicolini tribe, was included. All details of the taxa included in this study are listed in S1 Appendix.

### PCR Amplification and Sequencing

Four molecular markers were used in this study, the mitochondrial 16S and the nuclear 18S, 28S and Wingless (Wg). Amplification and sequencing were performed as previously described [2,11] and new sequences were deposited on GenBank (accession numbers KP263038—KP263055. Because we were able to use the most representative taxon sampling ever published for Triatominae [11], these molecular markers were chosen to include the greatest diversity of Reduviidae with the smallest possible number of missing taxa.

### Sequence Alignment

Alignment was performed individually for each of the markers. Ribosomal markers were aligned using MAFFT version 7 [22] under the Q-INS-i algorithm, which considers RNA secondary structure. The Wg marker was translated and then aligned using ClustalW [23] implemented on MEGA version 5 [24]. After being aligned, sequences were concatenated by name using SeaView version 4.2 [25]. Accordingly, the concatenated alignment comprised 237 taxa and 5928 base pairs (bp).

### Phylogenetic Reconstruction and Divergence Time Estimates

Phylogeny was reconstructed using both maximum likelihood (ML) [26] and Bayesian inference [27].

For the ML reconstruction, the concatenated alignment was analysed using PhyML 3.1 [28] under the GTR + G + I model with four gamma categories. The model with the largest number of parameters was chosen because insufficient parametrization can strongly enhance errors in the recovered tree [29]. This reconstruction was performed on the PhyML server [30]. This phylogeny was then used as the initial tree of a RaxML version 8 [31] search for 200 ML trees in independent runs. This time, the dataset was partitioned according to marker, and the GTR + G + I model was set to be estimated individually for each partition. Branch supports were estimated using the rapid bootstrap algorithm implemented on RAxML. This analysis was performed on the CIPRES Science Gateway [32]. The resulting best tree was then compared with the topologies obtained using a Bayesian approach.

Bayesian inference to estimate phylogeny and branching times simultaneously was performed using Beast version 1.8 [33], also on CIPRES. The analyses run under the GTR + G + I model with four gamma categories and calibration priors were also set as described below. The MCMC ran for 200,000,000 generations or until convergence was observed. The trees removed as burn-in represented 25% of the trees sampled.

To cross-validate the node ages obtained, the following three different reconstructions were performed using Beast: (1) one calibration point, in the diversification of Reduviidae. This single calibration was made because deeper calibrations can generate more accurate time estimates than shallower ones [34]; (2) six calibration points, restricting the minimum age of Emesinae to 25 Myr, based on the age of *Paleoploiariola venosa*; (3) seven calibration points, including *P*. *venosa*, as listed in Table 1.

**Table 1:** Description of the age priors used in Bayesian inference for time diversification. * Prior distribution used in the analysis that included *Paleoploiarola* venosa; #Prior distribution used in the analysis that did not include *Paleoploiarola venosa*. *Triatoma dominicana* has the same estimated age as *Panstrongylus hispaniolae*. Therefore, *Panstrongylus hispaniolae* was the only Triatominae fossil included.

FAMILY	SUBFAMILY	GENUS	SPECIES	LOCALITY	AGE (Ma)	Ingroup	Prior Distribution	Mean	StDev	REFERENCE
Ceresopseidae		*Ceresopsis*	*costalis*	Sogyuty, Kyrgyzstan	174–201 (Early cretacious)	Reduviidae	normal	185	10	[35]
Reduviidae	Phymatinae	*Koenisbergia*	*herczeki*	Samland Peninsula, Russia	33.9–56 (Eocene)	*Lophocustus* sp; *Macrocephalus* spp; *Phymata* spp.	normal	45	7	[36]
	Holoptilinae	*Praecoris*	*dominicana*	La Toca Mine, Dominican Republic	30–45 (Midlle Lutetian, Eocene to Middle Rupelian, Oligocene)	*Ptilonemus femoralis*; *Ptilocerus* sp	normal	37	5	[37]
	Emesinae	*Danzigia*	*christelae*	Samland Peninsula, Russia	33.9–56 (Eocene)	*Emesaya incisa; Stenolemus sp; Stenolemoides arizonensis; Mangabea barbiger; Ploiaria hirticornia; Empicoris sp*.	normal*	45	7	[36]
							exponential^#^	10,5	offset 25	
		*Paleoploiariola*	*venosa*	Cordillera Septentrional, Dominican Republic	25–40 (Late Oligocene to Bartonian, Eocene)	*Ploiaria hirticornia*; *Empicoris* sp.	normal	32,5	5	[38]
	Triatominae	*Panstrongylus*	*hispaniolae*	La Toca Mine, Dominican Republic	30–45 (Midlle Lutetian, Eocene to Middle Rupelian, Oligocene)	*Panstrongylus spp*; *T*. *dimidiata*; *T*. *longipennis*; *T*. *mazzottii*; *T*. *mexicana*; *T*. *pallidipennis*; *T*. *phyllosoma*; *T*. *picturata*; *T*. *ryckmani*; *T*. *bruneri*; *T*. *rubrofasciata*; *T*. *barberi*; *T*. *protracta*; *T*. *gerstaeckeri*; *T*. *lecticularia*; *T*. *recurva*; *T*. *rubida*; *T*. *sanguisuga*; *Linshcosteus sp*; *Pa*. *hirsuta*	normal	37	5	[21]
	Harpactorinae	*Apicrenus*	*fossilis*	La Toca Mine, Dominican Republic	30–45 (Midlle Lutetian, Eocene to Middle Rupelian, Oligocene)	*Arilus cristatus; Acanthiscium* sp	normal	37	5	[39]

### Phylogenies and Time Estimation Comparison

Four topologies were obtained and, to evaluate the discrepancies among them, the phylogenies were compared pairwise using TOPD/FMTS [40]. The algorithm used, namely, Disagree, shows not only the number of discordant nodes but also the taxa that are discordant. The comparison between the different topologies, obtained through independent runs, is a highly important step in the interpretation of the results because a greater degree of agreement among the results shows that the results should be more highly probable.

To compare the uncertainties of the age estimates obtained by each of the reconstructions, we plotted a linear regression for each node to relate the width (‘w’) of the 95% confidence interval for the node to the mean age of the node (‘m’). This graph compares all the ages obtained in the three Bayesian reconstructions, and the regression was calculated using R [41].

### Ancestral Area Reconstructions

The ancestral area distribution was estimated only for the Triatominae subtrees extracted from the ML and Bayesian topologies. The taxa distribution was coded according to either the collection site of the specimen or the known distribution of the species [3]. The distribution reviewed by Galvão et al. [3] was used as the basis for our analysis because we are not aware of any new records that extend the species ranges furnished by this review, as described below.

The distributional ranges were set as the biogeographic Transition zones/subregions and Dominions of Latin America, as described by Morrone [42] as follows: (A) Mexican Transition Zone; (B) Antillean subregion, Brazilian subregion; (C) Mesoamerican Dominion; (D) Pacific Dominion; (E) Boreal Brazilian Dominion; (F) South Brazilian Dominion, Chacoan subregion; (G) South-eastern Amazonian Dominion; (H) Chacoan Dominion; (I) Paraná Dominion; (J) South American transition zone. Two more ranges were introduced based on the distribution of the species that do not occur in the zones defined by Morrone. These additional ranges are (K) Old World and (L) North America (except Mexico; S2 Appendix).

Two alternative methods were used to reconstruct the ancestral geographic ranges in all of the four subtrees obtained (topologies from ML and the three Bayesian phylogenies): Statistical dispersal vicariance analysis (S-DIVA) and Bayesian Binary MCMC (BBM), both implemented in the computer software Reconstruct Ancestral States in Phylogenies (RASP) [43]. Because there is no indication of the ancestral population distribution, we conducted the BBM reconstruction independently under the three options available (Custom, Null and Wide), as suggested by the RASP developers. Two independent runs of 50,000 generations were performed for each of the BBM reconstructions, and the empirical model was used for sampling. The ancestor range was set to be a maximum of 12 (*i*.*e*., possibly occurring in the whole extant Triatominae range). These reconstructions resulted in 16 possible routes of events leading to Triatominae diversification (four reconstructions for each of the four topologies).

The main goal of these reconstructions was to identify possible vicariant events. All the nodes indicating vicariant events were compared, and the events were considered most likely to have occurred only if (1) they were recovered in at least 50% of the reconstructions and (2) the clades were the same in all four topologies.

Given the events that met our criteria, we then observed the estimated age of the event and the “routes” reconstructed for the hypothetical populations of the given node. This approach was used to identify a starting point for the search for possible geological/climatic events that could have been vicariant.

### Geological Events Tested

We hypothesised and tested that the major geological changes in the Neotropics since the Eocene have played a role in the diversification of Triatominae. Therefore, the following events were tested: The effects of the Andean uplift in the Amazonian area, including (1) the formation of the Pebas system (23–10 Ma) in western Amazonia, when the rivers in the area started to flow towards the northwest; (2) the formation of the Acre system (10–7 Ma), which isolated Pan-Amazonia and allowed allopatric speciation [44]; (3) the formation of the connection between the Amazon and the Atlantic Forest, which would consist of a corridor from southern Brazil through the Chaco (23–10 Ma) [45]; (4) the GAARlandia (GAAR = Greater Antilles + Aves Ridge) land bridge, which may have formed a pathway between Northern South America and the Greater Antilles at the Eo-Oligocene boundary (34 Ma) for approximately 2 Myr [46]; (5) the period of biodiversity exchange resulting from the closure of the Isthmus of Panama (10–2.7 Ma); (6) the Florida and Gulf Coast inundations during the Miocene [47]; and (7) dispersal across the Bering Land Bridge during the Eocene [48].

In one specific case, the presence of Triatomini in the Antilles, the possibility of hitchhiking with the closely associated subfamily Capromyinae (Rodentia: Capromyidae) [49] whose arrival in the Antilles dates to the Mid-Miocene [50] was also tested.

The ages of the geological events were compared to the estimated tMRCA of the clades in question (*i*.*e*., posterior probability distribution from each of the three dated phylogenies) using R [41]. To summarise the ages of the tMRCA, we used TreeStat, which is part of the BEAST package [33].

In addition to the vicariant events identified by the ancestor reconstruction, we also tested the hypothesis proposed by Abad-Franch *et al*. [12], in which the authors cite the possible role of the most recent Andean uplift in the cladogenetic event that separated the *cis-* and *trans*-Andean groups of Rhodniini. We tested whether the event that formed the Pebas System (23–10 Ma) [44] could have influenced this cladogenesis. The closure of the Isthmus of Panama (10.1–2.76 Ma) and the most recent Andean uplift (5.3–2.6 Ma) were tested to determine whether they might have influenced the diversification within the *trans*-Andean group (*pallescens* group).

The ages of the fossil Triatominae *Triatoma dominicana* and *Panstrongylus hipaniolae* [20,21] were also compared with the estimated age of the node and related to the events that could possibly have introduced this lineage into the area of the Dominican Republic.

## Results

### Phylogenetic Reconstruction and Divergence Time Estimates

#### Phylogenetic reconstruction comparison

The dataset composed of 237 taxa and 5928 bp was used in the reconstruction of four phylogenetic trees (S3–S6 Appendixes), one under ML and the other three under a Bayesian framework, using nested priors, as described in the methodology. In a more didactic style, we will use the following terms to refer to the phylogenies: ML (maximum likelihood phylogeny), B1 (Bayesian phylogeny reconstructed only with the root fossil prior), B2 (Bayesian phylogeny reconstructed with six fossil priors) and B3 (Bayesian phylogeny reconstructed with seven fossil priors).

The four topologies were compared pairwise (Table 2), and most of the differences found were observed between methods (*i*.*e*., Bayesian x ML), with the comparison B3 x ML being the most divergent, with a divergence of 29 taxa. It is highly important to emphasise two aspects of these comparisons: (1) most of the taxa placed differently between the topologies compared shared the same MRCA with the two other closest taxa; and (2) the taxa that were placed differently but that did not share the same MRCA were still closely related to the same taxa (*i*.*e*., were recovered within the same clade). The different clades recovered usually had low branch support in at least one of the phylogenies.

**Table 2:** Taxa identified as divergent in each of the comparisons between topologies. Posterior probabilities (PP) and bootstrap (BP) values obtained for the clades in which the taxa were recovered are indicated. *indicates that the taxa did not share the MRCA with the other two closest species but still was recovered in the same clade, *i*.*e*., closely related to the same set of species.—indicates that the clade was not recovered in the given phylogeny. ML (maximum likelihood phylogeny), B1 (Bayesian inference with one calibration point), B2 (Bayesian inference with six calibration points), B3 (Bayesian inference with seven calibration points).

**B2 x B1**	**PP (B2)**	**PP (B1)**
*Acanthaspis* sp.2	0.57	0.98
*Censorinus ferrugineus*	0.17	0.26*
*Ctenotrachelus* sp.	0.36	0.42
*Ectomocoris* sp.	0.99	0.51
*Inara flavopicta* 00052170	0.57	0.2
*Inara flavopicta* 00052191	0.99	0.2
*Noualhierana furtiva* 00218966	0.17	0.67*
*Noualhierana furtiva* CW224	0.39	0.81*
*Panstrongylus geniculatus*	0.92	0.61*
*Platymeris biguttata*	0.39	0.54*
*Poecilosphodrus gratiosus*	0.12	0.31
*Rasahus thoracicus*	0.46	0.51
*Triatoma carcavalloi*	0.75	0.33
*Triatoma dimidiata* 94	0.33	0.46
*Triatoma gerstaeckeri*	0.35	0.46
*Triatoma klugi*	0.19	0.33
*Triatoma mexicana*	0.33	0.67
*Triatoma rubrovaria*	0.19	0.77
*Velinus* sp.	0.12	0.19
**B3 x B1**	**PP (B3)**	**PP (B1)**
*Acanthaspis* sp.2	0.21	0.98
*Ctenotrachelus* sp. 00000181	0.31	0.42
*Inara flavopicta* 00052170	0.98	0.2
*Inara flavopicta* 00052191	0.21	0.2
*Noualhierana furtiva* 00218966	0.29	0.67*
*Noualhierana furtiva* CW224	0.47	0.81*
*Panstrongylus geniculatus*	0.93	0.61*
*Platymeris biguttata*	0.23	0.54*
*Poecilosphodrus gratiosus*	0.16	0.31
*Triatoma carcavalloi*	0.37	0.33
*Triatoma costalimai* 42	0.29	0.21
*Triatoma dimidiata*	0.34	0.46
*Triatoma gerstaeckeri*	0.32	0.46
*Triatoma guasayana* 54	0.21	0.21
*Triatoma klugi*	0.75	0.33
*Triatoma mexicana*	0.34	0.67
*Triatoma rubrovaria*	0.37	0.77
*Triatoma williami*	0.21	0.24
**B3 x B2**	**PP (B3)**	**PP (B2)**
*Acanthaspis* sp.2	0.21	0.57
*Ectomocoris* sp.	0.5	0.99
*Inara flavopicta* 00052170	0.98	0.57
*Inara flavopicta* 00052191	0.21	0.99
*Noualhierana furtiva* 00218966	0.29	0.17
*Platymeris biguttata*	0.23	0.39
*Poecilosphodrus gratiosus*	0.16	0.12
*Rasahus thoracicus*	0.5	0.46
*Triatoma carcavalloi*	0.37	0.75
*Triatoma costalimai*	0.29	0.21
*Triatoma guasayana*	0.21	0.21
*Triatoma klugi*	0.75	0.19
*Triatoma rubrovaria*	0.37	0.19
*Triatoma williami*	0.21	0.25
**ML x B1**	**BP (ML)**	**PP (B1)**
*Acanthaspis* sp.	91	0.5
*Acanthaspis* sp.2	95	0.98
*Corythucha* sp.	100	0.98
*Ctenotrachelus* sp.	46	0.42
*Ectomocoris* sp.	100	0.51
*Inara flavopicta* 00052170	95	0.2
*Inara flavopicta* 00052191	100	0.2
*Kodormus bruneosus*	34	0.93
*Nabis apicalis*	93	1
*Noualhierana furtiva* 00218966	27	0.67
*Oligotylus carneatus*	-	0.87
*Panstrongylus geniculatus*		0.61
*Panstrongylus lignarius*	18	0.83
*Paredocla chevalieri*	49	0.41
*Phallospinophylus setosus*	-	1
*Platymeris biguttata*	32	0.54
*Plynoides* sp.	63	0.5
*Poecilosphodrus gratiosus*	18	0.31
*Rasahus thoracicus*	61	0.51
*Tapeinus* sp. 00052200	67	0.67
*Tapeinus* sp. CW2009CW183	24	0.82
*Triatoma bruneri*	12	0.15
*Triatoma costalimai* 42	32	0.21
*Triatoma dimidiata*	30	0.46
*Triatoma guasayana* 54	62	0.21
*Triatoma guasayana* 84	64	0.8
*Triatoma jurbergi*	22	0.98
*Triatoma matogrossensis*	22	1
*Triatoma vandae*	44	1
**B2 x ML**	**PP (B2)**	**BP (ML)**
*Acanthaspis sp*.	0.47	91
*Corythucha sp*.	0.95	100
*Ctenotrachelus sp*.	0.36	46
*Nabis apicalis*	0.99	93
*Noualhierana furtiva 00218966*	0.17	27
*Noualhierana furtiva CW224*	0.39	10
*Oligotylus carneatus*	0.88	-
*Panstrongylus lignarius*	0.99	18
*Paredocla chevalieri*	0.41	49
*Phallospinophylus setosus*	1	-
*Platymeris biguttata*	0.39	32
*Plynoides sp*.	0.47	63
*Poecilosphodrus gratiosus*	0.12	
*Tapeinus sp*. *00052200*	0.59	67
*Tapeinus sp*. *CW2009CW183*	0.89	24
*Triatoma bruneri*	0.89	12
*Triatoma carcavalloi*	0.75	58
*Triatoma costalimai 42*	0.21	
*Triatoma gerstaeckeri*	0.35	20
*Triatoma guasayana 54*	0.21	62
*Triatoma guasayana 84*	0.79	64
*Triatoma jurbergi*	0.97	22
*Triatoma klugi*	0.19	58
*Triatoma matogrossensis*	1	22
*Triatoma rubrovaria*	0.19	38
*Triatoma vandae*	1	44
***B3 x ML***	**PP (B3)**	**BP (ML)**
*Acanthaspis sp*.	0.48	91
*Acanthaspis sp*. *2*	0.21	95
*Censorinus ferrugineus*	0.66	18
*Corythucha sp*.	0.96	100
*Ctenotrachelus sp*.	0.31	46
*Ectomocoris sp*.	0.5	100
*Inara flavopicta 00052170*	0.98	95
*Inara flavopicta 00052191*	0.21	100
*Nabis apicalis*	0.99	93
*Noualhierana furtiva 00218966*	0.29	27
*Noualhierana furtiva CW224*	0.47	10
*Oligotylus carneatus*	0.87	-
*Panstrongylus lignarius*	0.99	18
*Paredocla chevalieri*	0.4	49
*Phallospinophylus setosus*	1	-
*Platymeris biguttata*	0.23	32
*Plynoides sp*.	0.48	63
*Rasahus thoracicus*	0.5	61
*Tapeinus sp*. *00052200*	0.67	67
*Tapeinus sp*. *CW2009CW183*	0.83	24
*Triatoma bruneri*	0.9	12
*Triatoma carcavalloi*	0.37	58
*Triatoma gerstaeckeri*	0.32	20
*Triatoma guasayana 54*	0.21	62
*Triatoma guasayana 84*	0.78	64
*Triatoma jurbergi*	0.97	22
*Triatoma klugi*	0.75	58
*Triatoma matogrossensis*	1	22
*Triatoma rubrovaria*	0.37	38
*Triatoma vandae*	1	44

**Fig 1:**
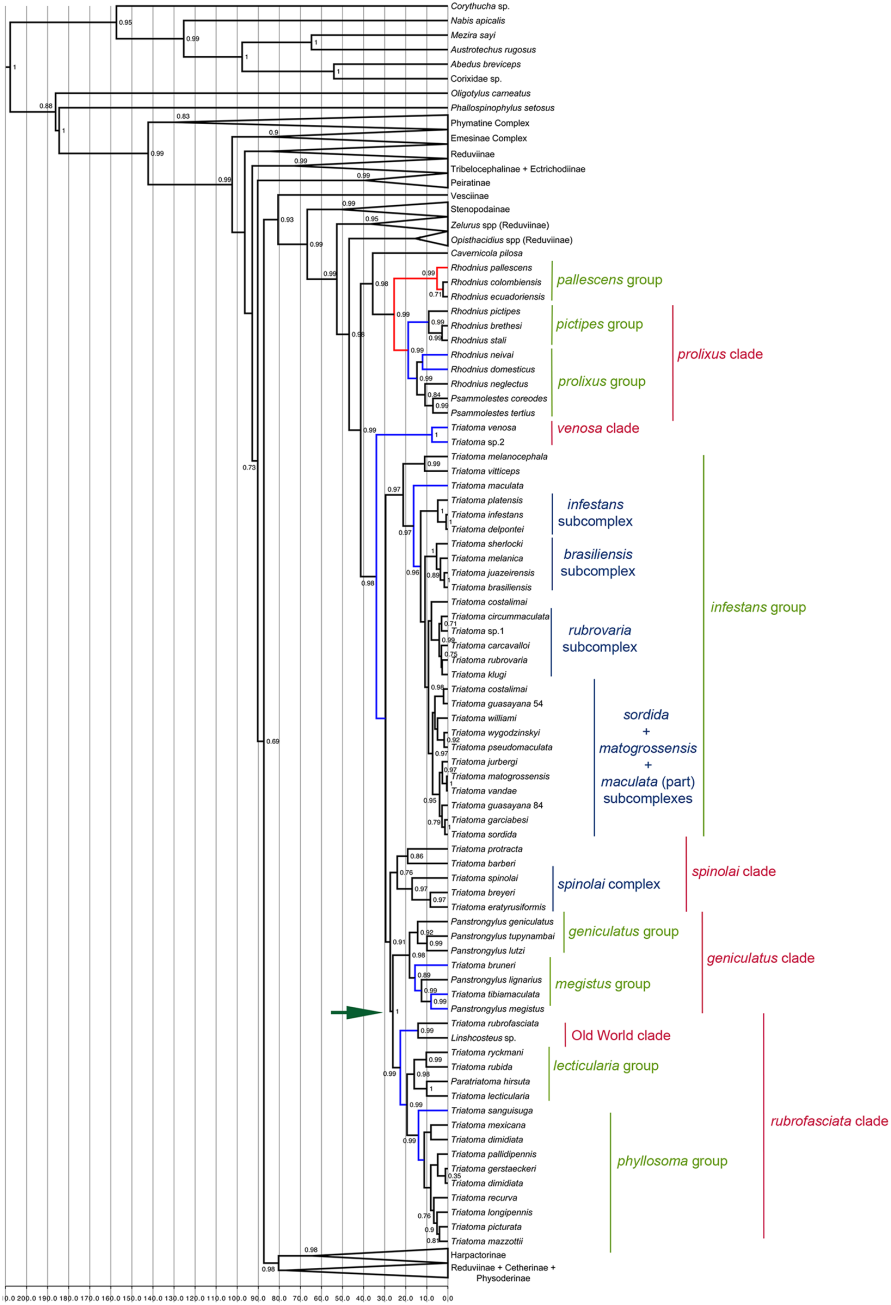
Bayesian phylogeny obtained with six fossil calibrations (B2). Numbers above branches indicate posterior probabilities greater than 0.50. Red branches indicate the cladogenetic events tested based on the literature. Blue branches indicate vicariant events recovered in our analyses and tested. Green arrow indicates the Triatominae fossil calibration used.

Most non-Triatomini Reduviidae relationships were recovered in concordance with those shown by Hwang & Weirauch [2], and, as they are not the focus of this study, they will not be discussed here. Because the comparison between B2 and B3 was the one that yielded the fewest differences, especially within Triatominae, B2 will be used to further discuss topology- related results (Fig 1).

Because the two most diverse genera in Triatomini are paraphyletic, we divided the tribe into clades > groups > complexes > subcomplexes for the purpose of this study. The complexes and subcomplexes are as in Schofield & Galvão [6].

Triatominae was recovered as monophyletic in all the reconstructions, with Cavernicolini as sister tribe to Rhodniini and these two tribes as sister to Triatomini. The Rhodniini tribe was recovered with the *trans*-Andean *Rhodnius* group (*pallescens* group) as sister to the *cis*-Andean *Rhodnius* clade (*pictipes* + *prolixus* groups). The genus P*asammolestes* was recovered within the clade comprising the *prolixus* group. For the Triatomini tribe, the first to diverge was the *venosa* clade. The *infestans* group (*infestans*, *brasiliensis*, *rubrovaria*, *sordida*, *matogrossensis* and *maculata* subcomplexes of *Triatoma*) was then recovered as sister to the remaining Triatomini.

#### Time estimation comparison

**Fig 2:**
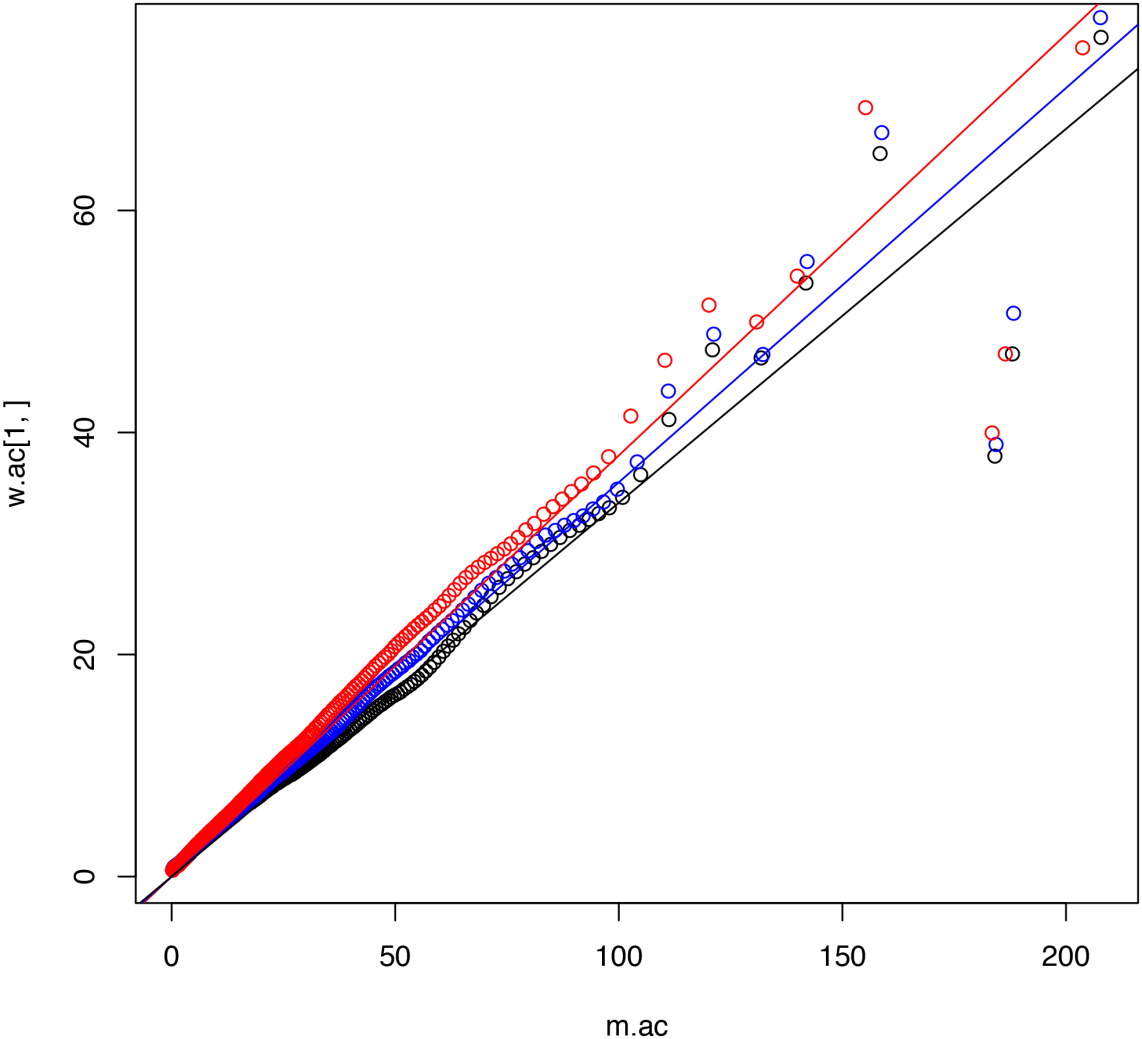
Time estimates and linear regression for the three Bayesian estimates. Red dots and line refer to the estimates using one fossil calibration (B1); blue dots and line refer to the estimates using six fossil calibrations (B2); black dots and line refer to the estimates using seven fossil calibrations (B3); Y axis represents the width (delta time interval) of the interval estimated and X axis the mean age estimated for each node in the phylogeny.

The estimated time for all the nodes obtained on the reconstructed B1, B2 and B3 were compared. Age means and confidence interval width were plotted, and a linear regression was calculated to (1) visualise the average ages estimated in the each of the reconstructions and (2) determine whether the precision of the estimates would increase with the number of calibrations. The results show that, despite the varying number of calibration priors, the three Bayesian time estimates yielded very similar ages for the diversification events and additionally, as expected, the accuracy of the estimates increased with the number of calibration points (Fig 2).

### Ancestral Area Reconstruction

To infer the origin of the ancestral population of the Triatominae, the ancestral area was reconstructed based on the current distribution of the subfamily. As we allowed the possible ancestral range to be the entire extant distribution, the parsimony analyses (S-DIVA) assigned the most probable distribution for the ancestral population as the entire current distribution for the four phylogenies analysed.

The other nine reconstructions, based on a Bayesian framework, showed otherwise. These reconstructions indicate that the ancestral population most likely lived in South America, more specifically in northern South America or even Central America (Pacific dominion) [42].

The reconstruction also indicated possible vicariant and dispersal events based on the divergence of Triatominae. In this case, our criterion for “most probable” status was that (1) the events should be recovered in more than 50% of the reconstructions and (2) the clade should be the same across the four phylogenies obtained. The event routes, including dispersal and vicariant events in these nodes, are detailed in S7 Appendix.

### Geological Events and Tests of the Timing of Cladogenesis

**Fig 3:**
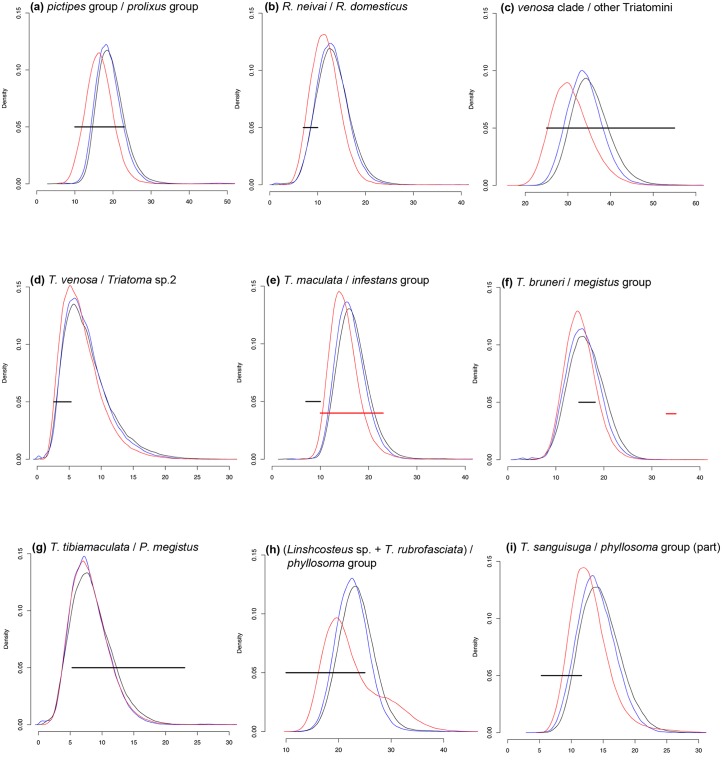
Vicariant events (black and red horizontal lines) plotted against posterior distribution (95% HPD) estimated for the nodes. (a) *pictipes* group / *prolixus* group–Pebas System; (b) *R*. *neivai* / *R*. *domesticus*–Acre System; (c) *venosa* clade / other Triatomini–Western Cordillera uplift; (d) *T*. *venosa* / *Triatoma* sp. 2 –last Andean uplift; (e) *T*. *maculata* / *infestans* group–Pebas System (red horizontal line) and Acre System (black horizontal line); (f) *T*. *bruneri* / *megistus* group–GAARlandia (red horizontal line) and rafting with Capromyinae (black horizontal line); (g) *T*. *tibiamaculata* / *P*. *megistus*–old Amazonian-Atlantic Forest pathway; (h) (*Linshcosteus* sp. + *T*. *rubrofasciata*) / *phyllosoma* group–Bering Land Bridge; (i) *T*. *sanguisuga* / *phyllosoma* group (part)–high sea level. Red distribution line refers to the estimates using one fossil calibration (B1); blue distribution line refers to the estimates using six fossil calibrations (B2); black distribution line refers to the estimates using seven fossil calibrations (B3). The vicariant events tested and the corresponding geological events are the following: (a) *pictipes* group / *prolixus* group–Pebas System; (b) *R*. *neivai* / *R*. *domesticus*–Acre System; (c) *venosa* clade / other Triatomini–Western Cordillera uplift; (d) *T*. *venosa* / *Triatoma* sp. 2 –last Andean uplift; (e) *T*. *maculata* / *infestans* group–Pebas System and Acre System; (f) *T*. *bruneri* / *megistus* group–GAARlandia and Rafting with Capromyinae; (g) *T*. *tibiamaculata* / *P*. *megistus*–old Amazonia-Atlantic Forest pathway; (h) (*Linshcosteus* sp. + *T*. *rubrofasciata*) / *phyllosoma* group–Bering Land Bridge; (i) *T*. *sanguisuga* / *phyllosoma* group (part)–high sea level (Fig 3a–3i and Table 3).

To test whether the vicariant events indicated by the reconstruction of the ancestral area matched the geological events observed in the distribution areas of the recovered clades in the estimated time period, we plotted the posterior distribution of the node ages against known ages of geological events in the area of interest (Fig 3). The analyses recovered nine vicariant events that met our testing criteria.

**Fig 4:**
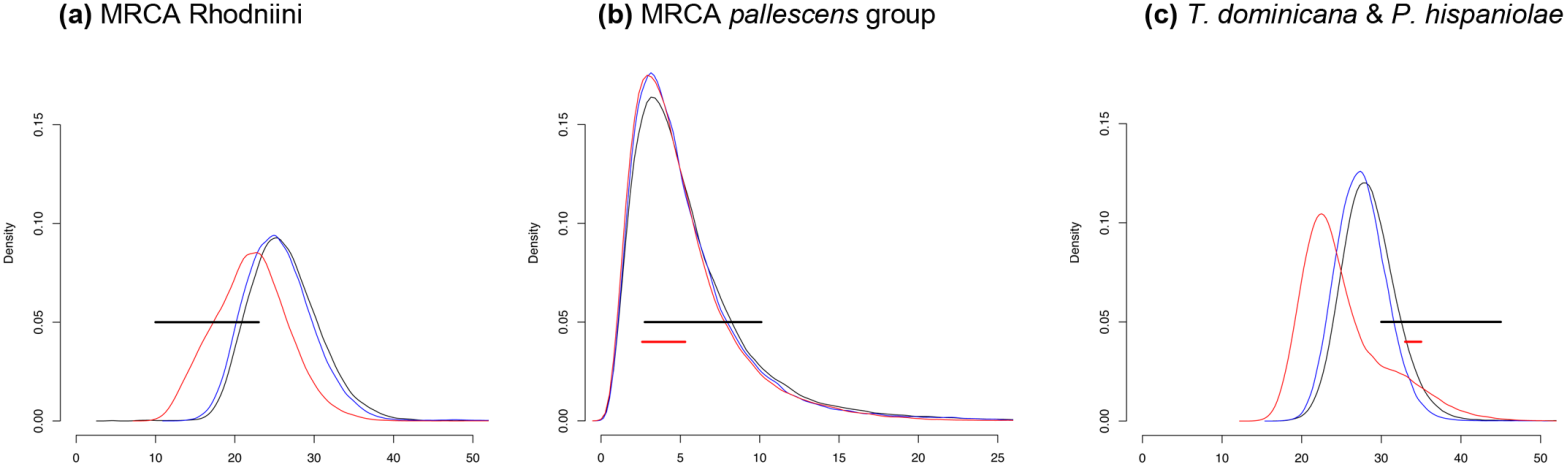
Other hypothesis testing. Events (black and red horizontal lines) plotted against posterior distribution (95% HPD) estimated for the nodes. Red distribution line refers to the estimates using one fossil calibration (B1); blue distribution line refers to the estimates using six fossil calibrations (B2); black distribution line refers to the estimates using seven fossil calibrations (B3); (a) Rhodniini MRCA–Pebas System; (b) *pallescens* group MRCA–Isthmus of Panama (red horizontal line) and last Andean uplift (Pliocene—black horizontal line); (c) (*spinolai* clade + *geniculatus* clade + *rubrofasciata* clade) estimated ages—Fossils *Triatoma dominicana* and *Panstrongylus hispaniolae* ages (black horizontal line) and GAARlandia land bridge (red horizontal line).

In addition, we tested one hypothesis previously proposed in the literature: that the Pebas system [44] would have separated the *cis*- and *trans*-Andean Rhodniini groups [12]. We also tested whether the most recent Andean uplift and the closure of the Isthmus of Panama influenced diversification within the *pallescens* group (Fig 4a and 4b and Table 3), even though these events were not recovered as vicariant in our analyses.

**Table 3:** Cladogenetic events tested in this study and the hypothetical vicariant events tested. / indicates the vicariant event identified and tested. Colums B1-3 indicate the 95% confidence interval estimated in Bayesian inference.

Cladogenetic event	Interval (95%)			Geological/Climatic event (null hypothesis)	Geological Event age (Ma)	Null hypoyhesis testing	Geological Event reference
	B1	B2	B3				
*R*. *pallescens* / (*R*. *ecuadoriensis* + *R*. *colombiensis)*	0.64–11.75	0.66–11.59	0.61–12.65	Panama isthmus	10.1–2.76	not rejected	[51,52]
				last Andean uplift (Pliocene)	5.3–2.6	not rejected	[44]
*pallescens* group / *(pictipes* group+ *prolixus* group)	12.33–30.53	17.76–34.16	18.15–34.86	Pebas System	23–10	not rejected	[44]
*pictipes* group / *prolixus* group	9.41–22.94	12.77–25.62	13.16–26.69	Pebas System	23–10	not rejected	[44]
*R*. *neivai* / *R*. *domesticus*	5.46–16.01	6.48–17.88	6.56–18.49	Acre System	10.0–7.0	not rejected	[44]
*venosa* clade / other Triatomini	22.23–39	26.25–41.75	23.96–37.99	Western Cordillera Uplift	55–25	not rejected	[62]
*T*. *venosa* / *Triatoma* sp.2	1.6–13.14	2.19–14.54	2.22–15.42	last andean uplift (Pliocene)	5.3–2.6	not rejected	[44]
*T*. *maculata* / *infestans* group	9.51–20.88	14.76–28.37	8.83–18.27	Pebas System	23–10	not rejected	[44]
				Acre System	10.0–7.0	rejected	[44]
*T*. *bruneri* / *megistus* group	8.14–19.72	9.29–22.29	9.22–23.16	Hitchhiking	14.8–18.2	not rejected	[50]
				GAARlandia	35–33	rejected	[46]
*T*. *tibiamaculata* / *P*. *megistus*	2.92–13.8	3.01–13.87	2.77–14.81	old AF/Am pathway	23–5.3	not rejected	[45]
(*Linshcosteus* sp. + *T*. *rubrofasciata*) / *phyllosoma* group	11.92–31.64	16.71–28.34	14.18–26.05	Bering land bridge	25–10	not rejected	[48]
*T*. *sanguisuga* / *phyllosoma* group (part)	5.94–15.59	8.41–19.73	6.87–16.9	high sea level in early Miocene	11.6–5.3	not rejected	[64]

We tested 13 geological events for the 11 mentioned cases of cladogenesis. The null hypothesis (geological event influencing diversification) was rejected for only two of these events: the Acre system as the vicariant event separating *T*. *maculata* from the *infestans* group (Fig 3e) and the GAARlandia land bridge as the route by which *T*. *bruneri* reached the Antillean islands (Fig 3f).

The node age, for which we used the fossils of *P*. *hispaniolae* and *T*. *dominicana* as the calibration prior, was estimated to be slightly younger that the age of the fossils but still within the confidence interval. Additionally, the GAARlandia land bridge could not be rejected as a possible route by which the lineage originating *P*. *hispaniolae* and *T*. *dominicana* reached the Antilles (Fig 4c).

## Discussion

The results of this study contributed new information to the study of the evolution of the vectors of Chagas Disease and to the further investigation of a long-debated question: whether Triatominae is monophyletic. We found that most of the diversification events observed within the Rhodniini and Triatomini were closely linked to the climatic and geological changes caused by the Andean uplift in South America and that sea level variations in North America also played a role in the diversification of the *Triatoma* species in that region.

### Triatominae Monophyly vs. Paraphyly

In the first major review of Triatominae systematics, Lent and Wygodzinsky [49] classified the subfamily as monophyletic, primarily based on characteristics related to haematophagy. Later, based on similarities to other Reduviidae, the suggestion of a paraphyletic origin for Triatominae arose [51] and has been discussed ever since [51–54].

Despite the extensive discussions of Triatominae systematics by those authors, the first thoroughly sampled cladistic analysis of the Reduviidae did not appear until 2008 [1], and the corresponding molecular phylogeny [55] was published a year later. Both the sampling and the reconstructions recovered Triatominae as monophyletic. Later, the inclusion of a greater diversity of Reduviinae (Hemiptera: Reduviidae) led to the recovery of a paraphyletic Triatominae and to the knowledge of their closest relatives [2].

Our results support the monophyletic hypothesis. We added several Triatominae taxa to the dataset published by Hwang & Weirauch [2] and recovered Triatominae as monophyletic in every phylogenetic reconstruction that we ran.

It is now fairly clear that a phylogeny is a hypothesis based on the data on hand, *i*.*e*., taxa and characters. Adding or removing taxa from a dataset may change the relationships between the remaining taxa, although extensive studies have shown that increasing diversity may increase phylogenetic accuracy if a sufficient number of characters are included for the taxa [56].

We now have a clearer scenario for the closest relatives of Triatominae. Based on this scenario, we can focus on performing further cladistic, morphological, phylogenetic and physiological studies including those relatives to better understand the nature of the ancestor or ancestors of Triatominae.

### Rhodniini

The Rhodniini tribe consists of two genera, the most diverse and paraphyletic *Rhodnius* and *Psammolestes*. Previous molecular phylogenetic studies agree with our results showing that *Psammolestes* groups within the clade formed by the *prolixus* group, one of the three *Rhodnius* groups, others being the *pallescens* and *pictipes* groups [10,57,58].

*The trans-Andean* Rhodnius *clade (*pallescens *group)—*Although the status of *Psammolestes* as a genus within the *prolixus* group is not contested, the relationships among *Rhodnius* groups are a different matter. In addition to the previous disagreement about the relationships among the three *Rhodnius* groups [10,57,58], Abad-Franch *et al*. [12] added to the controversy by assuming the *pallescens* (*trans*-Andean) group to be sister taxon to the *pictipes* (*cis*-Andean) group even though their area cladograms indicated a different scenario. The authors also suggested a vicariant event in the diversification of these groups: “*The ancestors of the lineage probably dispersed across the northern part of the (then low) Eastern Cordillera of the Colombian Andes in the Miocene*, *and became isolated with the subsequent rapid uplift ~ 5 million years ago*”. Accordingly, we tested their hypothesis.

First, our results indicate the *pallescens* group to be sister to the *prolixus* clade, which is consistent with the other two most diverse Triatominae phylogenies [10,11] and with the cladogram areas published by Abad-Franch *et al*. [12]. In addition, the *pallescens* group appears to have diverged much earlier than 5 Ma, when the Northern Andean uplift led to the formation of the Pebas system (23–10 Ma; Fig 4a) [44]. Most likely, the last Andean uplift appears to have influenced diversification within the group, separating *R*. *pallescens* from *R*. *colombiensis* + *R*. *ecuadoriensis*. This separation could also be a consequence of the dispersal of *R*. *pallescens* to Central America through the then-closed Panama isthmus or of both events combined (Fig 4b).

#### The cis-Andean Rhodnius clade (prolixus clade)

Our results show that the *cis*-Andean Rhodniini clade is monophyletic. This conclusion is in agreement with previously published phylogenies [10,11]. The novelty here is that our reconstruction of the ancestral area indicated a vicariant event separating the *pictipes* group from the *prolixus* group.

The comparison between the age of this event and the uplift of the northern Andes, which caused a scenario change in the Amazon termed the Pebas system [44], showed that this event can not be rejected as vicariant (Fig 1a). Most likely, the *pictipes* ancestor was isolated in the areas designated as the Chacoan subregion [42] and the *prolixus* ancestor in the Brazilian subregion [42]. The *pictipes* ancestor then broadened its range with the expansion of the Amazon in the new pathways between the Amazon and the Atlantic forests [45], reaching the current distributions of the species.

Another interesting possible vicariant event identified in our analyses is the one that led to the separation of *R*. *neivai* and *R*. *domesticus*. The morphological similarity between these species was previously observed by Lent and Wygodzinsky [49], as shown by the placement of these species next to each other in the identification key to the genus.

Because the Pebas system could not be discarded as the vicariant event leading to the diversification of the *prolixus* clade, we tested the next large event in the Amazon to understand if it would be the vicariant for these species. In that context, the appearance of the Acre system [44] cannot be rejected as a potential vicariant event (Fig 1b). Accordingly, based on the route predicted by our analyses, the population that represented the origin of *R*. *neivai* must have been isolated farther north (in the current Pacific and Boreal Brazilian Dominions), and the ancestor of *R*. *domesticus* must have been isolated in the South (current Chacoan and Panama Dominions).

### Triatomini

The recovered relationships within Triatomini generally agree with the previously published phylogenies [2,10,11,59] except for the position of the *spinolai* complex. In our results, instead of being recovered as a sister taxon to the South American *Triatoma*, it was always recovered such that it was more closely related to the *geniculatus* and *rubrofasciata* clades.

#### South American Triatoma

The Andean uplift had a major influence on the diversification of the Triatomini tribe, starting from the beginning of the uplift of the Western Cordillera (55–25 Ma) [60]. According to our analyses, the uplift of the Western Cordillera acted as a vicariant event separating the *venosa* clade from the remaining Triatomini (Fig 3c).

Subsequently, the Northern Andean uplift (23–10 Ma) separated *T*. *maculata* (restricted to the Amazon) from the other members of the *infestans* group except for *T*. *melanocephala* + *T*. *vitticeps*, which appear to have reached the Atlantic coast by dispersal and diversified prior to that event.

While the northern Andean uplift was active in the middle Miocene (14 Ma), the Acre system [44] formed a sea that covered an extensive area within the continent, with the Guiana and Brazilian Shields as islands. The ancestral population of *T*. *maculata* must have been restricted to the Guiana shield, while the population that diversified into the remaining subcomplexes in the *infestans* groups must have been isolated in the Brazilian shield.

The climatic changes observed as a consequence of the rapid Andean uplift (14 Ma) [61], would have resulted in the rapid diversification of isolated populations in the diagonal dry corridor (Caatinga through Chaco), originating the extant *brasiliensis* subcomplex in the Caatinga Province, the *rubrovaria* subcomplex in the Pampean Province, the *infestans* subcomplex in the Chacoan Province and the *sordida*, *matogrossensis* and *maculata* (part) subcomplexes in the Cerrado Provinces (groups assigned as in Schofield & Galvão 2009; Provinces as in Morrone 2014).

#### The geniculatus clade

One event, the arrival of the Triatomini in the Antilles, can be observed in this clade and will be further discussed later. The other event of interest produced the separation of the most intriguing pair of sister species of Triatomini: *T*. *tibiamaculata* and *P*. *megistus*.

Both *T*. *tibiamaculata* and *P*. *megistus* exhibit classic morphological features of each of their genera. However, no matter what molecular marker(s) are used to reconstruct phylogenies including these species and no matter what diversity level is considered, they are recovered as sister taxa with high support [10,11,59]. Our hypothesis, based on the reconstruction of the ancestral range, is that the ancestral population was distributed along the old connection between the Amazonian Forest and the Atlantic Forest [45]. With the climatic changes caused by the Andean uplift, this connection disappeared, leaving the dry corridor and acting as a vicariant event that originated the species we now know as *T*. *tibiamaculata* and *P*. *megistus*.

#### The rubrofasciata clade

In the next section, we discuss the possible route that Triatomini took to the Old World. Here, we will focus on our hypothesis about the event that produced the separation of *T*. *sanguisuga* from the other members of the *phyllosoma* group. In view of the current distribution of the lineages and the estimated age and distribution of the MRCA to this clade, we hypothesised that the most likely vicariant event here would be the high sea level in the early Miocene (11,6–5.3 Ma) that inundated Florida and the Gulf Coast, an event that also influenced the diversification of the Equinae (Mammalia: Perisodactyla) [62].

It is possible to observe that *T*. *dimidiata* s.l. was not recovered as a monophyletic species group. The fact that *T*. *dimidiata* is composed of more than one lineage is clear now [63,64], and the fact that this lineage is not monophyletic is supported by reproductive isolation in breeding experiments [65].

Due to limited sampling, we were not able to test if the formation of the Baja California peninsula would actually have influenced the separation of the three *T*. *rubida* subspecies [66], although our age estimate does not allow us to discard this hypothesis. *The arrival of Triatomini in non-continental American land areas and in the Old World—*Because Triatominae is a group that is distributed almost exclusively in the continental Americas, the question of their mode of dispersal to other land areas and to the Old World is natural and, to date, unanswered. It has been suggested that mice infested with Triatomini were carried to the Old World on ships, as is known for the current worldwide dispersal of *T*. *rubrofasciata* [67].

Our results show that the separation of the Old World clade, which we believe to be monophyletic [67], dates to a time as late as the Mid-Oligocene. In the same period (25–10 Ma), the Bering Land Bridge was a route for terrestrial animals as well as plants that migrated between North America and Asia. Warm and wet climate conditions, as found in that area during that period [48], appear to facilitate the dispersal of Triatomini. The analyses also indicated a vicariant event in the separation of this clade. We believe that this event was the disappearance of the land bridge after the dispersal of the ancestral population of the Old World Triatomini.

To examine the colonisation of the Antillean islands by Triatomini, we tested the hypothesis that the GAARlandia land bridge, which connected northern South America to the Antilles was a potential dispersal route for Triatomini. Although our calibration prior was set in a conservative way (*i*.*e*., on the tMRCA of the clade including all the specimens that showed similar morphological characters), the estimated age of the node is a little younger than the fossil age itself, although there is still superposition between the fossil age and B2 and B3 estimated ages. In addition, the age of the GAARlandia land bridge does not allow us to reject it as a possible route for those lineages to have arrived in the Antilles (Fig 4c).

One interesting finding is that our results also support a second arrival of Triatominae in the Antilles. Our dated phylogeny supports a more recent arrival based on the phylogenetic position recovered for *T*. *bruneri*. Although little information is available on this species, Lent & Wygodzinsky [49] described the closely related *T*. *flavida* as associated with a rodent species, *Capromys pilorides*. The rodent subfamily Capromyinae arrived in the Antilles approximately 14.8–18.8 Ma [50], at approximately the same time that the *T*. *bruneri* ancestor arrived. Therefore, as the species cited in this context are associated ecologically, it is reasonable to assume that whatever route (Fabre and his co-workers suggested rafting [45]) brought the Capromyinae to the Antilles, the *T*. *bruneri* ancestor “tagged along”.

## Supporting Information

S1 Appendix GenBank access list of the sequences used in this study, along with the taxonomic list and molecular marker identification. (DOCX)

S2 Appendix Distribution of the taxa used in this study according to Galvão et al. (2003) in the biogeographic provinces as defined by Morrone (2014). "1"indicates presence, "0" indicates absence of the given taxon in the geographic area. (A) Mexican Transition Zone; (B) Antillean subregion, Brazilian subregion; (C) Mesoamerican Dominion; (D) Pacific Dominion; (E) Boreal Brazilian Dominion; (F) South Brazilian Dominion, Chacoan subregion; (G) South-eastern Amazonian Dominion; (H) Chacoan Dominion; (I) Paraná Dominion; (J) South American transition zone; (K) Old World and (L) North America (except Mexico).
(DOCX)

S3 Appendix Maximum likelihood estimates using GTR+G+I and 100 bootstrap replicates. Numbers above branches represent bootstrap values greater than 50.
(DOCX)

S4 Appendix Bayesian phylogeny obtained using GTR+G+I with only the root fossil calibration (B1). Numbers above branches indicate posterior probabilities greater than 0.50. Blue bars indicate 95% HPD.
(DOCX)

S5 Appendix Bayesian phylogeny obtained using GTR+G+I with six fossil calibrations (B2). Numbers above branches indicate posterior probabilities greater than 0.50. Blue bars indicate 95% HPD.
(DOCX)

S6 Appendix Bayesian phylogeny obtained using GTR+G+I with seven fossil calibrations (B3). Numbers above branches indicate posterior probabilities greater than 0.50. Blue bars indicate 95% HPD.
(DOCX)

S7 Appendix Dispersal route and vicariant events identified in the analyses. Areas are coded as in Appendix 2. -> and ^ refer to dispersal events and | to vicariant events. BBM (null ancestor distribution), BBM (wide ancestor distribution) and BBM (custom ancestor distribution) are the ancestral distribution options for BBM inference.
(DOCX)
