# Supplementary material for: Geological Changes of the Americas and their Influence on the Diversification of the Neotropical Kissing Bugs (Hemiptera: Reduviidae: Triatominae)
Source: PLoS Negl Trop Dis. 2016 Apr 8;10(4):e0004527. doi: 10.1371/journal.pntd.0004527 (PMC4825970; doi:10.1371/journal.pntd.0004527)
Supplement: S1 Appendix — (DOCX) [file pntd.0004527.s001.docx]

**S1 Appendix:** GenBank access list of the sequences used in this study, along with the taxonomic list and molecular marker identification.

| **Family** | **Subfamily** | **Species** | **Marker** | | | | |
| --- | --- | --- | --- | --- | --- | --- | --- |
|  |  |  | **16S** | **28S** | **28S** | **18S** | **WG** |
| Belostomatidae | Belostomatinae | *Abedus breviceps* | AY252676 |  | AY252440 | AY252186 |  |
| Corixidae |  | *Corixidae* sp. | FJ230383 | FJ230537 | FJ230615,FJ230694 | FJ230456 | JQ897872 |
| Scutellaridae |  | *Austrotichus rugosus* | AY252745 |  | AY252517 | AY252171 |  |
| Aradidae | Mezirinae | *Mezira sayi* | EU683100 |  | EF641177 | AY252222 |  |
| Nabidae | Nabinae | *Nabis apicalis* | EF487292 |  | EF487339 | EF487316 |  |
| Tingidae |  | *Corythuca* sp. | JQ897789 | FJ230536 | FJ230614, FJ230693 | FJ230455 |  |
| Miridae | Phylinae | *Oligotylus carneatus* | AY252853 |  | AY252596 | AY252377 |  |
|  |  | *Phallospinophylus setosus* | FJ230382 | FJ230535 | FJ230613, FJ230692 | FJ230454 |  |
| Reduviidae | Centrocnemidinae | *Neocentrocnemis stali* |  | GU188466 | GU188447 | JQ897578 |  |
|  | Cetherinae | *Cethera musiva* | JQ897787 | JQ897629 | JQ897706 |  |  |
|  |  | *Cethera musiva* | JQ897788 | JQ897630 | JQ897707 | JQ897552 |  |
|  |  | *Eupheno histrionicus* | JQ897795 | JQ897636 | JQ897712 | JQ897556 | JQ897883 |
|  |  | *Eupheno pallens* | JQ897796 | JQ897637 | JQ897713 | JQ897557 |  |
|  | Ectrichodiinae | *Cleptria corallina* | FJ230388 | FJ230543 | FJ230621, FJ230700 | FJ230462 | JQ897871 |
|  |  | *Ectrichodia lucida* | FJ230387 | FJ230542 | FJ230620, FJ230699 | FJ230461 | JQ897878 |
|  |  | *Microsanta* sp. | JQ897793 | JQ897634 | JQ897710 |  | JQ897880 |
|  |  | *Ectrychotes* sp. |  | FJ230560 | FJ230638, FJ230717 | FJ230479 |  |
|  |  | *Ectrychotes* sp. | FJ230424 | FJ230584 | FJ230661, FJ230740 | FJ230503 | JQ897879 |
|  |  | *Maraenaspis* sp. | FJ230389 | FJ230544 |  | FJ230463 | JQ897889 |
|  |  | *Racelda* sp. | FJ230398 | FJ230553 | FJ230631, FJ230710 | FJ230472 | JQ897915 |
|  |  | *Rhiginia* sp. | FJ230410 | FJ230571 | FJ230648, FJ230727 | FJ230490 | JQ897917 |
|  | Emesinae | *Emesaya incisa* | FJ230436 | FJ230598 | FJ230672, FJ230751 | FJ230515 | JQ897881 |
|  |  | *Empicoris* sp. |  | FJ230567 |  | FJ230486 |  |
|  |  | *Mangabea barbiger* | FJ230441 | FJ230602 | FJ230674, FJ230753 |  | JQ897888 |
|  |  | *Ploiaria hirticornis* |  | FJ230556 | FJ230634, FJ230713 | FJ230475 |  |
|  |  | *Stenolemoides arizonensis* | FJ230444 | FJ230605 | FJ230677, FJ230756 | FJ230522 | JQ897923 |
|  |  | *Stenolemus* sp. | FJ230413 | FJ230573 |  |  |  |
|  | Holoptilinae | *Ptilocerus* sp. | GU188453 | GU188467 | GU188448 | JQ897599 |  |
|  |  | *Ptilocnemus femoralis* | FJ230431 | FJ230591 | FJ230667, FJ230746 | FJ230509 |  |
|  | Hammacerinae | *Microtomus cinctipes* | FJ230411 |  | FJ230649, FJ230728 | FJ230491 |  |
|  |  | *Microtomus* sp. | FJ230395 |  | FJ230628, FJ230707 | FJ230469 |  |
|  | Harpactorinae | *Acanthiscium seminigrum* | FJ230450 | FJ230607 | FJ230685, FJ230764 | FJ230530 |  |
|  |  | *Agriocoris flavipes* |  | FJ230569 | FJ230646, FJ230725 | FJ230488 |  |
|  |  | *Apiomerus lanipes* | FJ230435 | FJ230597 | FJ230671, FJ230750 | FJ230514 | JQ897868 |
|  |  | *Apiomerus ochropterus* | FJ230393 | FJ230548 | FJ230625, FJ230704 | FJ230466 | JQ897869 |
|  |  | *Arilus cristatus* | FJ230402 | FJ230558 | FJ230636, FJ230715 | FJ230477 |  |
|  |  | *Castolus subinermis* | FJ230446 |  | FJ230681, FJ230760 | FJ230526 |  |
|  |  | *Coranus callosus* | FJ230433 | FJ230594 | FJ230669, FJ230748 | FJ230511 |  |
|  |  | *Euagoras* sp. | FJ230427 | FJ230587 | FJ230663, FJ230742 | FJ230505 |  |
|  |  | *Harpactorinae* sp. | FJ230425 | FJ230585 | FJ230662, FJ230741 | FJ230504 |  |
|  |  | *Heniartes putumayo* |  | FJ230609 | FJ230686, FJ230766 |  |  |
|  |  | *Micrauchenus lineola* | FJ230397 | FJ230552 | FJ230630, FJ230709 | FJ230471 |  |
|  |  | *Poecilosphodrus gratiosus* | FJ230429 | FJ230589 | FJ230665, FJ230744 | FJ230507 |  |
|  |  | *Pselliopus spinicollis* | FJ230438 | FJ230600 |  | FJ230517 |  |
|  |  | *Pselliopus zebra* | FJ230434 | FJ230596 | FJ230670, FJ230749 | FJ230513 |  |
|  |  | *Pyrrhosphodrus amazonus* | FJ230396 | FJ230551 | FJ230629, FJ230708 | FJ230470 |  |
|  |  | *Rhaphidosoma decorsei* | FJ230390 | FJ230545 | FJ230622, FJ230701 | FJ230464 |  |
|  |  | *Rhynocoris segmentarius* | FJ230384 | FJ230538 | FJ230616, FJ230695 | FJ230457 |  |
|  |  | *Ricolla quadrispinosa* |  | FJ230610 | FJ230687 | FJ230531 |  |
|  |  | *Sinea diadema* | FJ230408 | FJ230566 | FJ230644, FJ230723 | FJ230485 |  |
|  |  | *Ulpius* sp *.* | FJ230449 |  | FJ230684, FJ230763 | FJ230529 |  |
|  |  | *Velinus* sp *.* | FJ230428 | FJ230588 | FJ230664, FJ230743 | FJ230506 |  |
|  |  | *Vesbius purpureus* | FJ230422 | FJ230582 | FJ230659, FJ230737 | FJ230501 |  |
|  |  | *Zelus longipes* | FJ230385 | FJ230539 | FJ230617, FJ230696 | FJ230458 |  |
|  |  | *Zelus renardii* |  | FJ230565 | FJ230643, FJ230722 | FJ230484 |  |
|  |  | *Zelus renardii* | FJ230453 |  | FJ230691, FJ230770 | FJ230534 |  |
|  | Peiratinae | *Ectomocoris atrox* | FJ230447 |  | FJ230682, FJ230761 | FJ230527 | JQ897876 |
|  |  | *Ectomocoris ornatus* |  | FJ230595 |  | FJ230512 | JQ897877 |
|  |  | *Peirates punctorius* | FJ230430 | FJ230590 | FJ230666, FJ230745 | FJ230508 | JQ897908 |
|  |  | *Rasahus thoracicus* |  |  | FJ230679, FJ230758 | FJ230525 | JQ897916 |
|  | Phymatinae | *Lophoscutus* sp. | FJ230400 | FJ230555 | FJ230633, FJ230712 | FJ230474 |  |
|  |  | *Macrocephalus barberi* | FJ230437 | FJ230599 | FJ230673, FJ230752 | FJ230516 |  |
|  |  | *Macrocephalus sp* | FJ230409 | FJ230568 | FJ230645, FJ230724 | FJ230487 | JQ897887 |
|  |  | *Phymata acutangula* | FJ230394 | FJ230550 | FJ230627, FJ230706 | FJ230468 |  |
|  |  | *Phymata fortificata* |  | FJ230549 | FJ230626, FJ230705 | FJ230467 | JQ897909 |
|  |  | *Phymata pacifica* | FJ230401 | FJ230557 | FJ230635, FJ230714 | FJ230476 |  |
|  |  | *Phymata* sp. | FJ230407 | FJ230564 | FJ230642, FJ230721 | FJ230483 | JQ897910 |
|  | Physoderinae | *Physoderes impexa* | JQ897830 | JQ897662 | JQ897748 | JQ897591 | JQ897911 |
|  |  | *Physoderes vestita* | JQ897831 | JQ897663 | JQ897749 | JQ897592 | JQ897912 |
|  |  | *Physoderes* sp. | JQ897832 | JQ897664 |  | JQ897593 | JQ897913 |
|  | Reduviinae | *Acanthaspis bilineolata* | JQ897773 |  | JQ897690 | JQ897540 | JQ897859 |
|  |  | *Acanthaspis gulo* | JQ897774 | JQ897619 | JQ897691 |  | JQ897860 |
|  |  | *Acanthaspis iracunda* | FJ230392 | FJ230547 | FJ230624, FJ230703 |  | JQ897861 |
|  |  | *Acanthaspis iracunda* | JQ897775 |  | JQ897692 | JQ897541 | JQ897862 |
|  |  | *Acanthaspis laosensis* | JQ897776 |  | JQ897693 |  |  |
|  |  | *Acanthaspis bimaculata* | JQ897777 |  | JQ897694 | JQ897542 | JQ897863 |
|  |  | *Acanthaspis quadriannulata* | JQ897778 | JQ897620 | JQ897695 |  | JQ897864 |
|  |  | *Acanthaspis sulcipes* | JQ897781 |  | JQ897698 | JQ897545 | JQ897866 |
|  |  | *Acanthaspis westermanni* | JQ897782 |  | JQ897699 | JQ897546 | JQ897867 |
|  |  | *Acanthaspis* sp. | JQ897779 | JQ897621 | JQ897696 | JQ897543 |  |
|  |  | *Acanthaspis* sp. | FJ230403 | FJ230559 | FJ230716 | FJ230478 | JQ897865 |
|  |  | *Acanthaspis* sp. |  | FJ230716 | FJ230637 | FJ230478 |  |
|  |  | *Acanthaspis* sp. | JQ897780 | JQ897622 | JQ897697 | JQ897544 |  |
|  |  | *Acanthaspis sp. 2* | FJ230406 | FJ230720 | FJ230641, FJ230563 | FJ230482 |  |
|  |  | *Alloeocranum arboricolum* | JQ897783 | JQ897623 | JQ897700 | JQ897547 |  |
|  |  | *Censorinus ferrugineous* | JQ897786 | JQ897628 | JQ897705 | JQ897551 |  |
|  |  | *Durevius tuberculatus* | JQ897790 | JQ897631 | JQ897708 | JQ897553 |  |
|  |  | *Durganda rubra* | JQ897791 | JQ897632 |  |  | JQ897874 |
|  |  | *Dyakocoris vulnerans* | JQ897792 | JQ897633 | JQ897709 | JQ897554 | JQ897875 |
|  |  | *Gerbelius ornatus* | JQ897797 |  | JQ897714 | JQ897558 |  |
|  |  | *Gerbelius ornatus* | JQ897799 |  | JQ897716 | JQ897560 |  |
|  |  | *Gerbelius ornatus* | JQ897798 | JQ897638 | JQ897715 | JQ897559 |  |
|  |  | *Gerbelius* sp. | JQ897800 | JQ897639 | JQ897717 |  |  |
|  |  | *Inara alboguttata* | JQ897801 | JQ897640 | JQ897718 | JQ897561 |  |
|  |  | *Inara flavopicta* |  |  | JQ897719 | FJ230482 |  |
|  |  | *Inara flavopicta* | JQ897802 | JQ897641 | JQ897720 | JQ897562 |  |
|  |  | *Kayanocoris wegneri* | JQ897803 | JQ897642 | JQ897721 | JQ897563 | JQ897884 |
|  |  | *Leogorrus immaculatus* | JQ897804 |  | JQ897722 | JQ897564 |  |
|  |  | *Leogorrus litura* | FJ230386 | FJ230540 | FJ230618, FJ230697 | FJ230459 |  |
|  |  | *Leogorrus litura* | JQ897805 |  | JQ897723 | JQ897565 | JQ897885 |
|  |  | *Leogorrus longiceps* |  | FJ230570 | FJ230647, FJ230726 | FJ230489 |  |
|  |  | *Leogorrus longiceps* | JQ897806 |  | JQ897724 | JQ897566 |  |
|  |  | *Leogorrus* n. sp. | JQ897807 |  | JQ897725 | JQ897567 | JQ897886 |
|  |  | *Microlestria fuscicollis* | JQ897808 | JQ897643 | JQ897726 | JQ897568 |  |
|  |  | *Microlestria fuscicollis* | JQ897809 | JQ897644 | JQ897727 | JQ897569 |  |
|  |  | *Microlestria fuscicollis* | JQ897810 |  | JQ897728 | JQ897570 |  |
|  |  | *Nalata spinicollis* | JQ897812 | JQ897646 | JQ897730 | JQ897572 |  |
|  |  | *Nalata setulosa* | JQ897813 |  | JQ897731 | JQ897573 | JQ897890 |
|  |  | *Nalata squalida* | JQ897815 | JQ897648 | JQ897733 | JQ897575 | JQ897892 |
|  |  | *Nalata* sp. | JQ897814 | JQ897647 | JQ897732 | JQ897574 | JQ897891 |
|  |  | *Nanokerala browni* | JQ897816 | JQ897649 | JQ897734 | JQ897576 |  |
|  |  | *Nanokerala browni* | JQ897817 | JQ897650 | JQ897735 | JQ897577 | JQ897893 |
|  |  | *Neostachyogenys tristis* | JQ897818 | JQ897651 | JQ897736 | JQ897579 |  |
|  |  | *Noualhierana furtiva* | FJ230432 | FJ230592 | FJ230668, FJ230747 | FJ230510 | JQ897894 |
|  |  | *Opisthacidius chinai* | JQ897819 | JQ897652 | JQ897737 | JQ897580 | JQ897896 |
|  |  | *Opisthacidius mexicanus* | JQ897820 | JQ897653 | JQ897738 | JQ897581 | JQ897897 |
|  |  | *Opisthacidius sp* | JQ897821 | JQ897654 | JQ897739 | JQ897582 | JQ897898 |
|  |  | *Paraplynus lugubris* | FJ230420 | FJ230580 | FJ230657, FJ230736 | FJ230499 |  |
|  |  | *Paredocla chevalieri* | FJ230391 | FJ230546 | FJ230623, FJ230702 | FJ230465 | JQ897902 |
|  |  | *Pasiropsis maculata* | JQ897825 | JQ897658 | JQ897743 | JQ897586 | JQ897903 |
|  |  | *Pasiropsis marginata* | JQ897826 |  | JQ897744 | JQ897587 | JQ897904 |
|  |  | *Pasiropsis* n. sp. | JQ897827 | JQ897659 | JQ897745 | JQ897588 | JQ897905 |
|  |  | *Pasiropsis* sp. | JQ897829 | JQ897661 | JQ897747 | JQ897590 | JQ897907 |
|  |  | *Pasiropsis* sp. | JQ897828 | JQ897660 | JQ897746 | JQ897589 | JQ897906 |
|  |  | *Platymeris biguttata* | FJ230418 | FJ230578 | FJ230655, FJ230734 | FJ230497 |  |
|  |  | *Plynoides* sp. | JQ897833 | JQ897665 | JQ897750 | JQ897594 |  |
|  |  | *Pseudozelurus arizonicus* | JQ897834 | JQ897666 | JQ897751 | JQ897595 |  |
|  |  | *Pseudozelurus superbus* | JQ897835 | JQ897667 | JQ897752 | JQ897596 |  |
|  |  | *Psophis* sp. | JQ897836 | JQ897668 | JQ897753 | JQ897597 | JQ897914 |
|  |  | *Psophis* sp. |  | JQ897669 | JQ897754 | JQ897598 |  |
|  |  | *Reduvius personatus* | JQ897837 |  |  | JQ897600 |  |
|  |  | *Reduvius sonoraensis* | JQ897838 |  |  |  |  |
|  |  | *Staliastes rufus* | JQ897842 | JQ897673 | JQ897758 | JQ897604 | JQ897921 |
|  |  | *Staliastes* sp. | JQ897843 | JQ897674 | JQ897759 | JQ897605 | JQ897922 |
|  |  | *Tiarodes versicolor* | JQ897847 | JQ897678 | JQ897763 | JQ897608 | JQ897927 |
|  |  | *Tapeinus* sp. | JQ897845 | JQ897676 | JQ897761 | JQ897606 | JQ897925 |
|  |  | *Tapeinus* sp. | FJ230421 |  | FJ230658, FJ230737 | FJ230500 | JQ897926 |
|  |  | *Tiarodes* sp. | JQ897846 | JQ897677 | JQ897762 | JQ897607 |  |
|  |  | *Varus flavoannulatus* | JQ897852 | JQ897683 | JQ897768 | JQ897613 |  |
|  |  | *Velitra rubropicta* | JQ897853 | JQ897684 | JQ897769 |  | JQ897933 |
|  |  | *Velitra* sp. | JQ897854 | JQ897685 | JQ897770 | JQ897614 | JQ897934 |
|  |  | *Zelurus alcides* | JQ897855 | JQ897686 | JQ897771 | JQ897615 | JQ897935 |
|  |  | *Zelurus petax* | FJ230416 |  | FJ230653, FJ230732 | FJ230495 |  |
|  |  | *Zelurus pintoi* | JQ897856 | JQ897687 |  | JQ897616 |  |
|  |  | *Zelurus* sp. | JQ897857 | JQ897688 | JQ897772 | JQ897617 | JQ897936 |
|  |  | *Zelurus* sp. | JQ897858 | JQ897689 |  | JQ897618 | JQ897937 |
|  |  | *Zelurus* sp. | FJ230412 |  | FJ230650, FJ230729 | FJ230492 |  |
|  | Saicinae | *Kiskeyana palassaina* |  | FJ230541 | FJ230619, FJ230698 | FJ230460 |  |
|  |  | *Saica* sp. | FJ230399 | FJ230554 | FJ230632, FJ230711 | FJ230473 |  |
|  | Salyavatinae | *Lisarda vandenplasi* | FJ230419 | FJ230579 | FJ230656, FJ230735 | FJ230498 |  |
|  |  | *Lisarda sp* | FJ230404 | FJ230561 | FJ230639, FJ230718 | FJ230480 |  |
|  |  | *Salyavatinae* sp. | JQ897841 | JQ897672 | JQ897757 | JQ897603 |  |
|  | Stenopodainae | *Canthesancus* sp. | JQ897784 | JQ897624 | JQ897701 |  | JQ897870 |
|  |  | *Ctenotrachelus* sp. | FJ230415 | FJ230575 | FJ230652, FJ230731 | FJ230494 | JQ897873 |
|  |  | *Gageus micropterus* | FJ230445 | FJ230606 | FJ230678, FJ230757 | FJ230524 |  |
|  |  | *Kodormus bruneosus* | FJ230452 |  | FJ230690, FJ230769 | FJ230533 |  |
|  |  | *Oncocephalus* sp. | FJ230405 | FJ230562 | FJ230640, FJ230719 | FJ230481 | JQ897895 |
|  |  | *Sastrapada* sp. | FJ230423 |  | FJ230660, FJ230739 | FJ230502 | JQ897920 |
|  |  | *Stenopoda* sp. | FJ230414 |  | FJ230651, FJ230730 | FJ230493 | JQ897924 |
|  |  | *Stenopodessa* sp. | FJ230451 |  | FJ230688, FJ230767 | FJ230532 |  |
|  |  | *Thodelmus nigrispinosus* | FJ230448 |  | FJ230683, FJ230762 | FJ230528 |  |
|  |  | *Stenopodainae* sp. | JQ897844 | JQ897760 | JQ897675 |  |  |
|  | Triatominae | *C. pilosa* | JQ897785 | JQ897704 | JQ897627 | JQ897550 |  |
|  |  | *P. tertius* | AY035439 |  |  | Y18751 |  |
|  |  | *P. coreodes* | AF045708 |  |  |  |  |
|  |  | *R. colombiensis* | AY035438 | KC543516 |  |  |  |
|  |  | *R. ecuadoriensis* |  | KC543518 |  |  |  |
|  |  | *R. pallescens* |  | KC543527 |  |  |  |
|  |  | *R. brethesi* | KC248980 |  |  |  |  |
|  |  | *R. pictipes* | JQ897840 | JQ897756 | JQ897671 | JQ897602 | JQ897919 |
|  |  | *R. stali* | KC248984 |  |  |  |  |
|  |  | *R. domesticus* | AY035440 |  |  |  |  |
|  |  | *R. neivai* | AY035441 |  |  |  |  |
|  |  | *R. prolixus* |  | AF435862 |  |  |  |
|  |  | *R.neglectus* | JQ897839 | JQ897755 | JQ897670 | JQ897601 | JQ897918 |
|  |  | *Linshcosteus sp.* | AF394595 | GQ853373 | GQ853372 | AJ421954 |  |
|  |  | *P. geniculatus* | JQ897822 | JQ897741 | JQ897740 | JQ897583 | JQ897899 |
|  |  | *P. lignarius* | JQ897823 | JQ89774 | JQ897656 | JQ897584 | JQ897900 |
|  |  | *P. lutzi* | KC248969 | KC249135 |  |  | KP263046 |
|  |  | *P. megistus* | KC248970 | KC249136 |  | AJ243336 |  |
|  |  | *P.tupynambai* | KC248978 | KC249142 |  |  |  |
|  |  | *Pa. Hirsuta* | FJ230443 | FJ230604 | FJ230676 | FJ230521 |  |
|  |  | *T. dimidiata* | KC249004 | KC249152 |  |  |  |
|  |  | *T. dimidiata* | KC249005 | KC249155 |  |  |  |
|  |  | *T. longipennis* | KC249031 | KC249177 |  | AJ243331 | KP263048 |
|  |  | *T. mazzottii* | AY035446 |  |  | AJ243333 |  |
|  |  | *T. mexicana* | JX872251 |  |  |  |  |
|  |  | *T. pallidipennis* | KC249044 | KC249184 |  | KC249115 |  |
|  |  | *T. picturata* | AY185840 |  |  | AJ243332 |  |
|  |  | *T. ryckmani* | JX872248 |  |  |  |  |
|  |  | *T. bruneri* | KC248989 | KC249146 |  |  | KP263049 |
|  |  | *T. rubrofasciata* | AY127046 |  |  | AJ421960 |  |
|  |  | *T. barberi* | JX872242 |  |  | AJ421958 |  |
|  |  | *T. protracta* | KC249048 | FJ230754 | FJ230675 | FJ230520 | JQ897929 |
|  |  | *T. gerstaeckeri* |  | KF188642 |  |  |  |
|  |  | *T. lecticularia* | KC249029 | KC249175 |  | KC249111 |  |
|  |  | *T. recurva* | FJ230417 | FJ230654 | FJ230577 | FJ230496 | JQ897930 |
|  |  | *T. rubida* | AY035445 | GQ853391 |  |  |  |
|  |  | *T. sanguisuga* | JX890269 | GQ853392 |  |  |  |
|  |  | *T. venosa* | JQ897850 | JQ897766 | JQ897681 | JQ897611 | JQ897932 |
|  |  | *T. brasiliensis* | KC248985 | KC249145 |  |  |  |
|  |  | *T. juazeirensis* | KC249026 | KC249173 |  |  |  |
|  |  | *T. melanica* | KC249041 | KC249183 |  |  |  |
|  |  | *T. sherlocki* | KC249068 | KC249205 |  |  |  |
|  |  | *T. delpontei* | KC249000 | KC249150 |  |  |  |
|  |  | *T. infestans* | KC249016 | KC249169 |  | KC249107 |  |
|  |  | *T. platensis* | KC249047 | KC249186 |  |  |  |
|  |  | *T. maculata* | KC249035 | KC249178 |  |  |  |
|  |  | *T. pseudomaculata* | KC249051 | KC249190 |  |  |  |
|  |  | *T. wygodzynski* | KC249090 | KC249222 |  | KC249133 |  |
|  |  | *T. costalimai* | KC248997 |  |  | KC249101 |  |
|  |  | *T. costalimai* | KC248998 | KC249149 |  |  |  |
|  |  | *T. jurbergi* | KC249027 | KC249174 |  | KC249110 | KP263047 |
|  |  | *T. matogrossensis* | KC249036 | KC249179 |  | KC249112 | KP263040 |
|  |  | *T. vandae* | KC249083 | KC249217 |  | KC249129 |  |
|  |  | *T. williami* | KC249089 |  |  |  |  |
|  |  | *T. carcavalloi* | KC248990 |  |  | KC249096 |  |
|  |  | *T. circummaculata* | KC248992 | KC249147 |  | KC249098 | KP263054 |
|  |  | *T. klugi* | KC249028 |  |  |  |  |
|  |  | *T. rubrovaria* | KC249067 | KC249204 |  | KC249122 | KP263042 |
|  |  | *T. garciabesi* | KC249006 | KC249158 |  | KC249102 | KP263038 |
|  |  | *T. guasayana* | KC249012 | KC249163 |  | KC249104 |  |
|  |  | *T. guasayana* | - |  |  |  | KP263039 |
|  |  | *T.sordida* | KC249073 | KC249208 |  | KC249124 |  |
|  |  | *T. breyeri* | KC248988 |  |  |  |  |
|  |  | *T. eratyrusiformis* | AY035466 |  |  |  |  |
|  |  | *T. spinolai* | AF324518 |  |  | AJ421961 |  |
|  |  | *T. melanocephala* | KF769450 |  |  |  |  |
|  |  | *T. tibiamaculata* | KC249080 | KC249214 |  | KC249127 | KP263055 |
|  |  | *T. vitticeps* | KC249086 | KC249219 |  | KC249131 |  |
|  |  | *Triatoma* sp.1 | KC249008 | KC249160 |  |  | KP263052 |
|  |  | *Triatoma* sp. 2 | JQ897849 | JQ897765 | JQ897680 | JQ897610 | JQ897931 |
|  | Tribelocephalinae | *Tribelocephala peyrierasi* | FJ230440 |  | FJ230601 | FJ230521 |  |
|  |  | *Tribelocephalinae* sp. | JQ897851 | JQ897767 | JQ897682 | JQ897612 |  |
|  | Vesciinae | *Mirambulus niger* | JQ897811 | JQ897729 | JQ897645 | JQ897571 |  |
|  | Visayanocorinae | *Carayonia orientalis* |  | JQ897703 | JQ897626 | JQ897549 |  |
|  |  | *Carayonia* n. sp. |  | JQ897702 | JQ897625 | JQ897548 |  |
