# Supplementary material for: Geological Changes of the Americas and their Influence on the Diversification of the Neotropical Kissing Bugs (Hemiptera: Reduviidae: Triatominae)
Source: PLoS Negl Trop Dis. 2016 Apr 8;10(4):e0004527. doi: 10.1371/journal.pntd.0004527 (PMC4825970; doi:10.1371/journal.pntd.0004527)
Supplement: S3 Appendix — Numbers above branches represent bootstrap values greater than 50. (DOCX) [file pntd.0004527.s003.docx]

**S3 Appendix:** Maximum likelihood estimates using GTR+G+I and 100 bootstrap replicates. Numbers above branches represent bootstrap values greater than 50.

**
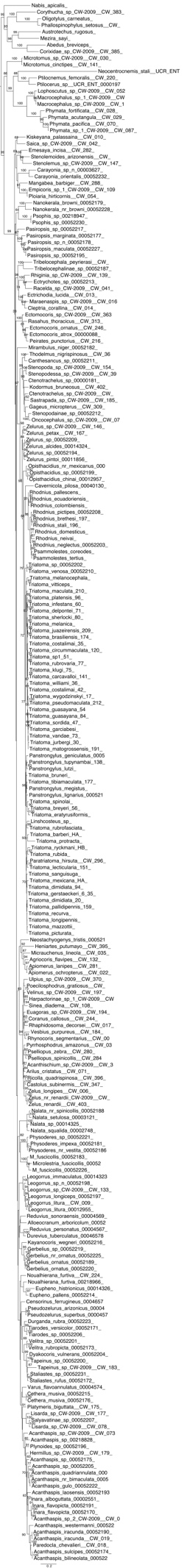
**
