# Supplementary material for: Geological Changes of the Americas and their Influence on the Diversification of the Neotropical Kissing Bugs (Hemiptera: Reduviidae: Triatominae)
Source: PLoS Negl Trop Dis. 2016 Apr 8;10(4):e0004527. doi: 10.1371/journal.pntd.0004527 (PMC4825970; doi:10.1371/journal.pntd.0004527)
Supplement: S5 Appendix — Numbers above branches indicate posterior probabilities greater than 0.50. Blue bars indicate 95% HPD. (DOCX) [file pntd.0004527.s005.docx]

**S5 Appendix:** Bayesian phylogeny obtained using GTR+G+I with six fossil calibrations (B2). Numbers above branches indicate posterior probabilities greater than 0.50. Blue bars indicate 95% HPD.

**
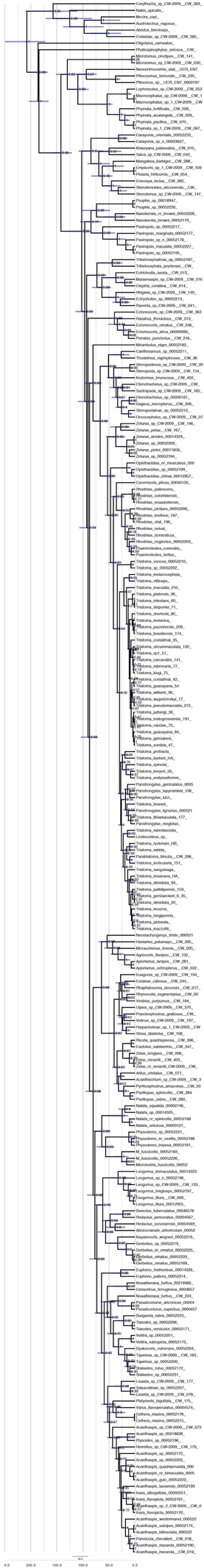
**
