# Supplementary material for: Geological Changes of the Americas and their Influence on the Diversification of the Neotropical Kissing Bugs (Hemiptera: Reduviidae: Triatominae)
Source: PLoS Negl Trop Dis. 2016 Apr 8;10(4):e0004527. doi: 10.1371/journal.pntd.0004527 (PMC4825970; doi:10.1371/journal.pntd.0004527)
Supplement: S7 Appendix — Areas are coded as in Appendix 2. -> and ^ refer to dispersal events and | to vicariant events. BBM (null ancestor distribution), BBM (wide ancestor distribution) and BBM (custom ancestor distribution) are the ancestral distribution options for BBM inference. (DOCX) [file pntd.0004527.s007.docx]

**S7 Appendix:** Dispersal route and vicariant events identified in the analyses. Areas are coded as in Appendix 2. -> and ^ refer to dispersal events and | to vicariant events. BBM (null ancestor distribution), BBM (wide ancestor distribution) and BBM (custom ancestor distribution) are the ancestral distribution options for BBM inference.

| **Cladogenetic event** | **ML** | | | |
| --- | --- | --- | --- | --- |
|  | **S-DIVA** | **BBM (null ancestor distribution)** | **BBM (wide ancestor distribution)** | **BBM (custom ancestor distribution)** |
| *pictipes* group / *prolixus* group | CEGHJ->HGJ->HDFGJ->H\|DFGJ | DEF->HICDEFG->HI\|CDEFG | DEF->HICDEFGJ->HI\|CDEFGJ | DEF->HICDEFG->HI\|CDEFG |
| *R. neivai* / *R. domesticus* | DHI->HIDE->HI\|DE | H->HIDE->HI\|DE | HI->HIDE->HI\|DE | H->HIDE->HI\|DE |
| *T. venosa* / *Triatoma* sp.2 | DE->D\|E | D->DE->D\|E | D->DE->D\|E | D->DE->D\|E |
| *T. maculata* / *infestans* group | DEH->DE\|H | HI->DEHI->DE\|HI | HI->DEHI->DE\|HI | HI->DEHI->DE\|HI |
| *venosa* clade / other Triatomini | ABCDEFHIKL->ABCDEFHIKL^E->ABCEFHIKL\|DE | D->HD->H\|D | H->HD->H\|D | D->HD->H\|D |
| (*T. barberi* + *T. protracta*) / *spinolai* complex |  |  |  |  |
| *T. bruner*i / *megistus* group | BF->B\|F | H->BH->B\|H | H->BH->B\|H | H->BH->B\|H |
| *T. tibiamaculata* / *P. megistus* | FHI->FH\|I | F->FHI->FH\|I | F->FHI->FH\|I | F->FHI->FH\|I |
| *(Linshcosteus* sp. + *T. rubrofasciata*) / *phyllosoma* group | ACHIKL->A\|CHIKL | H->H^L->ACLHK^L->ACL\|HKL | H->ALHK->AL\|HK | H->ALHK->AL\|HK |
| (*Pa. hirsuta* + *T. lecticularia*) / (*T. rubida* + *T. ryckmani*) | AL->L\|A | ACL->ACL^L->L\|ACL | AL->AL^L->LAC^L->L\|ACL | ACL->ACL^L->L\|ACL |
| *T. sanguisuga* / *phyllosoma* group (part) |  |  |  |  |
| *T. dimidiata* 20 / *T. gerstaecker*i |  |  |  |  |
| *R. pallescens* / (*R. ecuadoriensis* + *R. colombiensis*) | CDJ->CD\|J | D->D^D->CDJ^D->CD\|DJ | CDJ->CDJ^D->CD\|DJ | D->D^D->CDJ^D->CD\|DJ |
|  |  |  |  |  |
|  | **B1** | | | |
|  | **S-DIVA** | **BBM (null ancestor distribution)** | **BBM (wide ancestor distribution)** | **BBM (custom ancestor distribution)** |
| *pictipes* group / *prolixus* group | CFHJ->DHICEFJ->DHI\|CEFJ | DEF->HICDEFG->HI\|CDEFG | DEFIJ->DEFIJ^D^E^F^J->DEFIJ\|DEFJ | DEF->HICDEFG->HI\|CDEFG |
| *R. neivai* / *R. domesticus* | DHI->HIDE->HI\|DE | H->HIDE->HI\|DE | HI->HIDE->HI\|DE | H->HIDE->HI\|DE |
| *T. venosa* / *Triatoma* sp.2 | DE->E\|D | D->ED->E\|D | D->ED->E\|D | D->ED->E\|D |
| *T. maculata* / *infestans* group | DEH->DE\|H | HI->DEHI->DE\|HI | HI->DEHI->DE\|HI | HI->DEHI->DE\|HI |
| *venosa* clade / other Triatomini | ABDEFJKL->ABEJKLD->ABEJKLD^E->ABCEHIJKLD^E->ABCEHIJKL\|DE | D->HD->H\|D | H->HID->HI\|D | D->HD->H\|D |
| (*T. barberi* + *T. protracta*) / *spinolai* complex | - |  |  |  |
| *T. bruner*i / *megistus* group | BF->B\|F | H->->BF->B\|F | H->->BF->B\|F | H->->BF->B\|F |
| *T. tibiamaculata* / *P. megistus* | FHI->I\|FH | H->IFH->I\|FH | H->IFH->I\|FH | H->IFH->I\|FH |
| *(Linshcosteus* sp. + *T. rubrofasciata*) / *phyllosoma* group | ACIKL->LCIK->LCIK^L->L\|CIKL | L->LK->L\|K | L->L^L->LHK^L->L\|HKL | L->LHK->L\|HK |
| (*Pa. hirsuta* + *T. lecticularia*) / (*T. rubida* + *T. ryckmani*) | CL->L\|C | L->LAC->L\|AC | L->L^L->LAC^L->L\|ACL | L->L^L->LAC^L->L\|ACL |
| *T. sanguisuga* / *phyllosoma* group (part) |  |  |  |  |
| *T. dimidiata* 20 / *T. gerstaecker*i |  |  |  |  |
| *R. pallescens* / (*R. ecuadoriensis* + *R. colombiensis*) | DJ->DJ^D->CDJ^D->CD\|DJ | D->D^D->CDJ^D->CD\|DJ | CDJ->CDJ^D->CD\|DJ | D->D^D->CDJ^D->CD\|DJ |
|  |  |  |  |  |
|  | **B2** | | | |
|  | **S-DIVA** | **BBM (null ancestor distribution)** | **BBM (wide ancestor distribution)** | **BBM (custom ancestor distribution)** |
| *pictipes* group / *prolixus* group | CFHJ->CEFJDHI->CEFJ\|DHI | DEF->CDEFGHI->CDEFG\|HI | DEF->CDEFGHI->CDEFG\|HI | DEF->CDEFGHI->CDEFG\|HI |
| *R. neivai* / *R. domesticus* | DHI->DEHI->DE\|HI | H->DEHI->DE\|HI | H->DEHI->DE\|HI | H->DEHI->DE\|HI |
| *T. venosa* / *Triatoma* sp.2 | DE->D\|E | D->DE->D\|E | D->DE->D\|E | D->DE->D\|E |
| *T. maculata* / *infestans* group | DEH->DE\|H | HI->DEHI->DE\|HI | HI->DEHI->DE\|HI | HI->DEHI->DE\|HI |
| *venosa* clade / other Triatomini | ABDEFJKL->DEABJKL->DEABJKL^E->DEABCHIJKL^E->DE\|ABCEHIJKL | D->DH->D\|H | H->DH->D\|H | D->DH->D\|H |
| (*T. barberi* + *T. protracta*) / *spinolai* complex | AJ->A\|J | H->->AJ->A\|J | H->->AJ->A\|J | H->->AJ->A\|J |
| *T. bruner*i / *megistus* group | BF->B\|F | H->->BF->B\|F | H->->BF->B\|F | H->->BF->B\|F |
| *T. tibiamaculata* / *P. megistus* | FHI->I\|FH | F->IFH->I\|FH | F->IFH->I\|FH | F->IFH->I\|FH |
| *(Linshcosteus* sp. + *T. rubrofasciata*) / *phyllosoma* group | ACIKL->LCIK->LCIK^L->L\|CIKL | L->LHK->L\|HK | L->LHK->L\|HK | L->CLHK->CL\|HK |
| (*Pa. hirsuta* + *T. lecticularia*) / (*T. rubida* + *T. ryckmani*) | CL->CL^L->L\|CL | L->L^L->LAC^L->L\|ACL | L->L^L->LAC^L->L\|ACL | CL->CL^L->LAC^L->L\|ACL |
| *T. sanguisuga* / *phyllosoma* group (part) | AL->L\|A | L->L^L->LAC^L->L\|ACL | L->L^L->LAC^L->L\|ACL | L->L^L->LAC^L->L\|ACL |
| *T. dimidiata* 20 / *T. gerstaecker*i | ADL->LACD->L\|ACD | AC->LACD->L\|ACD | AC->LACD->L\|ACD | AC->LACD->L\|ACD |
| *R. pallescens* / (*R. ecuadoriensis* + *R. colombiensis*) | DJ->DJ^D->CDJ^D->CD\|DJ | D->D^D->CDJ^D->CD\|DJ | CDJ->CDJ^D->CD\|DJ | D->D^D->CDJ^D->CD\|DJ |
|  |  |  |  |  |
|  | **B3** | | | |
|  | **S-DIVA** | **BBM (null ancestor distribution)** | **BBM (wide ancestor distribution)** | **BBM (custom ancestor distribution)** |
| *pictipes* group / *prolixus* group | CFHJ->DHICEFJ->DHI\|CEFJ | DEF->HICDEFG->HI\|CDEFG | DEF->HICDEFGJ->HI\|CDEFGJ | DEF->HICDEFG->HI\|CDEFG |
| *R. neivai* / *R. domesticus* | DHI->DEHI->DE\|HI | DHI->DEHI->DE\|HI | HI->DEHI->DE\|HI | H->DEHI->DE\|HI |
| *T. venosa* / *Triatoma* sp.2 | DE->D\|E | DE->D\|E | D->DE->D\|E | D->DE->D\|E |
| *T. maculata* / *infestans* group | DEH->DE\|H | DEH->DE\|H | HI->DEHI->DE\|HI | HI->DEHI->DE\|HI |
| *venosa* clade / other Triatomini | ABDEFJKL->DEABJKL->DEABJKL^E->DEABCHIJKL^E->DE\|ABCEHIJKL | ABDEFJKL->DEABJKL->DEABJKL^E->DEABCHIJKL^E->DE\|ABCEHIJKL | H->DH->D\|H | D->DH->D\|H |
| (*T. barberi* + *T. protracta*) / *spinolai* complex | AJ->J\|A | AJ->J\|A | H->->JA->J\|A | H->->JA->J\|A |
| *T. bruner*i / *megistus* group | BF->B\|F | BF->B\|F | H->->BF->B\|F | H->->BF->B\|F |
| *T. tibiamaculata* / *P. megistus* | FHI->I\|FH | FHI->I\|FH | F->IFH->I\|FH | F->IFH->I\|FH |
| *(Linshcosteus* sp. + *T. rubrofasciata*) / *phyllosoma* group | ACIKL->CIKL->CIKL^L->CIKL\|L | ACIKL->CIKL->CIKL^L->CIKL\|L | L->HKCL->HK\|CL | L->HKCL->HK\|CL |
| (*Pa. hirsuta* + *T. lecticularia*) / (*T. rubida* + *T. ryckmani*) | CL->CL^L->CL\|L | L->L^L->ACL^L->ACL\|L | CL->CL^L->ACL^L->ACL\|L | L->L^L->ACL^L->ACL\|L |
| *T. sanguisuga* / *phyllosoma* group (part) | AL->L\|A | L->L^L->LAC^L->L\|ACL | L->L^L->LAC^L->L\|ACL | L->L^L->LAC^L->L\|ACL |
| *T. dimidiata* 20 / *T. gerstaecker*i | ADL->ACDL->ACD\|L | ADL->ACDL->ACD\|L | AC->ACDL->ACD\|L | AC->ACDL->ACD\|L |
| *R. pallescens* / (*R. ecuadoriensis* + *R. colombiensis*) | DJ->DJ^D->CDJ^D->CD\|DJ | D->D^D->CDJ^D->CD\|DJ | CDJ->CDJ^D->CD\|DJ | D->D^D->CDJ^D->CD\|DJ |
